# Impact of Alternative Feed Ingredients and Feeding Strategies on Growth, Muscle Morphology, and Fillet Quality of Genetically Selected Gilthead Seabream (*Sparus aurata*) in a Long-Term Feeding Trial

**DOI:** 10.3390/ani15131913

**Published:** 2025-06-28

**Authors:** Paula Sarmiento, Pedro L. Castro, Rafael Ginés

**Affiliations:** Aquaculture Research Group (GIA), Institute of Sustainable Aquaculture and Marine Ecosystems (IU-ECOAQUA), University of Las Palmas Gran Canaria, 35214 Telde, Spain; pedro.castro@ulpgc.es (P.L.C.); rafael.gines@ulpgc.es (R.G.)

**Keywords:** gilthead seabream, alternative diets, selective breeding, feeding strategy, fillet quality

## Abstract

Aquaculture advancement depends on alternative raw materials to reduce reliance on fishmeal (FM) and fish oil (FO) from extractive fisheries. Strategies like restricted feeding reduce costs and improve sustainability by minimizing feed waste and enhancing water quality, while selective breeding boosts growth and adapts fish to innovative diets. However, these measures may affect quality and shelf life. Gilthead seabream selected for high growth or non-selected were fed in 500 L tanks for 300 days until apparent satiety (AS) or with food restrictions (85AS or 65AS) using a control diet with low FM and FO balanced with vegetable ingredients, and an alternative diet (ALT) where FM was substantially replaced with insect, poultry by-product, feather, and porcine blood meals, while FO was completely replaced by microalgae, poultry, and salmon by-product oils. The ALT diet improved EPA + DHA levels, *n*-3/*n*-6 ratio, and fillet hardness. The selected fish outperformed the non-selected ones in growth and were more resilient to ALT diet and feeding restrictions. The 85AS feeding strategy optimized fillet quality by preventing lipid accumulation and muscle adaptations, as observed with the other feeding strategies. Combining sustainable feed formulations, genetic selection, and moderate feed restriction enables a viable, long-term strategy for high-quality, environmentally responsible seabream farming.

## Simple Summary

As aquaculture expands, its sustainability depends on strategies that improve resource efficiency. This study evaluates the long-term effects of replacing extractive marine-based ingredients with sustainable alternatives, combined with feeding restrictions and genetic selection in gilthead seabream (*Sparus aurata*). High-growth selected and non-selected fish were fed either to apparent satiety or under restricted feeding (85% and 65% of satiety). Two diets were tested: a control diet based on fishmeal and fish oil, and an alternative diet based on sustainable protein and oil sources. The results show that the alternative diet is not only feasible but also enhances fillet quality by improving the balance between omega-3 and omega-6 fatty acids and fillet firmness. The selected fish showed superior growth and adapted better to the alternative diet and feeding restrictions. Feeding at 85% satiety improved fillet quality by reducing lipid accumulation and avoiding muscle stress. These findings suggest that combining an alternative diet with novel sustainable ingredients, genetic selection, and moderate feed restriction is a promising strategy to improve sustainability and product quality of gilthead seabream farming.

## 1. Introduction

In recent years, there has been a focus on improving aquaculture sustainability, reducing its environmental impact [1,2]. New strategies are needed to deal with the limited resources of wild fish traditionally used to produce raw materials, such as fishmeal (FM) and fish oil (FO) [1,2]. Therefore, it is necessary to find new, profitable, and sustainable ingredients to replace FM and fish oil FO without compromising fish health and growth performance, especially in carnivorous marine fish such as the gilthead seabream (*Sparus aurata*; GSB), one of the most produced species in the Mediterranean, which represents an important economic resource with a growing demand [3].

Since the EU approved the use of insect meal (IM) for aquaculture (Regulation EU 2017/893), IM has become a promising alternative to FM due to its high protein and lipid content, efficient production, and alignment with the European Green Deal [4,5,6,7]. Similarly, microalgae oils (AOs) are a sustainable alternative to FO, rich in omega-3 long-chain polyunsaturated fatty acids (*n*-3LC-PUFA) content, which reduce the dependence on FO in feed for many farmed fish species, including GSB [8,9,10]. Animal by-products, such as poultry meal [11,12], feathermeal [12,13], blood meal [12,14], poultry oil (PO) [15,16], and salmon oil [15,17], are suitable ingredients to replace FM and FO in aquafeeds. The low price of animal by-products allows them to be used in combination with expensive ingredients, making aquafeed formulations more flexible [18]. Several studies have investigated replacing marine raw materials with alternative ingredients in GSB diets without negative effects during different growth stages [18,19,20]. Nonetheless, these studies are generally limited to juvenile fish. Long-term studies until commercial size are essential to assess whether early dietary effects persist or change over time [21,22]. Moreover, short-term or juvenile-stage trials overlook final fillet quality traits that directly impact consumer acceptance and market value [18,23].

Complementary strategies to optimize GSB production have been supported by genetic studies. Selective breeding programs aim to enhance growth rates and adaptability to innovative, low-FM/FO diets [24,25]. These nutritional challenges have been shown to enhance the ability of fish to utilize plant-based diets in future generations [26], although the results vary widely depending on species and study conditions [27,28].

Feeding strategies, such as satiation feeding or restricted rations, significantly influence the growth rate of farmed fish [29,30]. Restricted feeding offers environmental and economic benefits by improving water quality and reducing production costs related to feed and labor [31,32]. The highest feed efficiency is achieved at a feeding rate above the maintenance level, but below the maximal or satiation level [29,30]. Despite its potential, the long-term effects of dietary restriction on GSB are poorly understood.

It is important to emphasize that these strategies (alternative diets, selective breeding, and restricted feeding doses) can alter fillet nutrition, sensory traits, texture, and shelf life, directly impacting consumer acceptance [33,34]. Fish muscle undergoes rapid softening during post-harvest storage due to the proteolytic degradation of muscle fibers, significantly affecting fillet texture and quality. Understanding post-harvest muscle processes, such as proteolytic degradation, is economically crucial for maintaining fillet quality [35,36]. Immunohistochemical and ultrastructural analyses reveal microscopic changes in GSB muscle fibers due to enzymatic activity [37,38].

The present study evaluates the combined impact of multiple strategies aimed at enhancing the use of more sustainable feed formulations in GSB farming. Specifically, the study had three primary objectives: (1) to assess the long-term effects of replacing FM and FO with novel alternative ingredients; (2) to compare the responses of high-growth selected genotypes to those of reference (non-selected) genotypes; and (3) to assess the impact of different feeding strategies (restricted feeding vs. apparent satiety). These objectives were investigated throughout the entire on-growing period up to commercial size, a previously unexplored area, with additional attention given to growth performance, fillet composition, and quality during shelf life.

## 2. Materials and Methods

### 2.1. Ethical Statement

The feeding trial was conducted at the aquaculture facilities of ECOAQUA-UI, University of Las Palmas de Gran Canaria, Spain, following European Union Directive 2010/63/EU and Spanish legislation (RD 1201/2005) for animal protection in scientific research. All the procedures were approved by the Bioethical Committee of the University of Las Palmas de Gran Canaria (reference: OEBA-ULPGC-16-2021).

### 2.2. Experimental Diets

Two isoproteic and isoenergetic diets were used: a control (CTRL) diet with a low level of FM (15%) and FO (7%) balanced with vegetable meal (VM) (36.6%) and vegetable oil (VO) (7.7%), and an alternative (ALT) diet which replaced FM and VM with insect meal, poultry by-product meal, feathermeal hydrolysate, and porcine blood meal. Half of the VO and all FO were substituted with microalgae oil (AO), poultry oil (PO), and salmon by-product oils. The experimental diets were prepared at GIA, ECOAQUA, ULPGC, Spain. Each diet was extruded into 2 mm and 4 mm pellets to ensure physical stability and was formulated to sink slowly, facilitating appropriate feeding behavior and reducing feed losses. The diet formulation and proximate composition of the experimental diets are shown in Table 1.

### 2.3. Experimental Fish Rearing Trial and Sampling

The experimental fish came from two different broodstock groups included in the Spanish National Breeding Programme for GSB (PROGENSA^®^): high-growth (HG) fish from breeders selected based on the higher Estimated Breeding Value (+223.18), and reference (REF) fish from breeders with a lower Estimated Breeding Value (−159.14), considered as the reference population. Progeny (4th generation) from either selected fish for high-growth (HG) or reference fish (REF) was maintained under similar conditions during the larval pre-weaning, weaning, and early juvenile growth phases. Carvalho et al. [18] described the mating scheme of the genotypes HG and REF in detail. In total, 2700 GSBs with an initial body weight of 17.1 ± 1.8 g were randomly distributed in 36 tanks of 500 L with an initial and final density of 2.57 ± 0.03 kg/m^3^ and 13.60 ± 2.33 kg/m^3^, respectively. Fish were manually fed three times daily, and the amount of feed supplied as well as uneaten feed was weighed daily to calculate the amount of ingested feed. Three different feeding strategies were employed: feeding until apparent satiety (AS), feeding at 85% apparent satiety (85AS), and feeding at 65% apparent satiety (65AS). Each experimental group (genotype × diet × feeding strategy) was tested in triplicate (twelve experimental groups). The feeding period was 300 days. Tanks were randomly assigned to treatments using a randomized block design, and technicians performing biometric and fillet quality assessments were blinded to treatment allocation to reduce potential bias.

Throughout the experiment, the fish were kept under natural photoperiod (12 h light: 12 h dark), the average water temperature was maintained at 21.07 ± 1.68 °C, and the level of dissolved oxygen was consistently measured at 6.5 ± 0.5 mg/L.

The fish were sampled for growth every 60 days to check the proper condition and development of the fish. Before samplings, the fish were fasted for 24 h. All the fish were anesthetized with clove oil (0.2 mL/L). At the end of the trial, when the fish reached the commercial size, 10 fish per tank were slaughtered, simulating commercial conditions according to UNE 173300 [39], and packed with flaked ice in polystyrene boxes stored at 4 °C, replacing ice as necessary. Raw fillet samples were collected for texture, biochemical, and histological analyses, including ultrastructure, at 1 and 4 days post-harvest (dph), while fillets for sensory analysis were only collected at 1 dph.

### 2.4. Fish Growth and Feed Utilization

Productive parameters related with growth performance and feed utilization, including the condition index (K), specific growth rate (SGR), feed conversion ratio (FCR), and feed intake (FI), were calculated according to the following formulae: survival (%) = (final fish number/initial fish number) × 100; condition index K = Weight/Length^3^ × 100; specific growth rate SGR (%) = (Ln W1 − Ln W0)/days × 100; feed conversion ratio FCR = Ingested food/biomass generated; feed intake FI (g/fish/day) = fish food intake (g)/days, where W0: initial body weight (g) and W1: final body weight (g).

### 2.5. Fillet Quality

#### 2.5.1. Biochemical Analysis

GSB unskinned fillets (n = 15) on days 1 and 4 dph were individually homogenized prior to analysis. Proteins, lipids, and moisture were quantified by Near-InfraRed spectroscopy (NIR) of FoodScan^TM^ (FOSS, Hillerød, Denmark) [40]. The ash content was determined by incineration of the samples using a muffle furnace (Carbolite, Hope, UK) at 600 °C until a constant weight was achieved [41]. For fatty acid analyses, the lipids were extracted with chloroform/methanol (2:1 *v*/*v*) [42], transmethylated to obtain fatty acid methyl esters [43], separated by gas–liquid chromatography [44], quantified using a flame ionization detector (Agilent Technologies, Santa Clara, CA, USA), and identified by comparison with external and well-characterized standards (SUPELCO 37 FAME MIX, Ref. CRM47885, Supelco, Bellefonte, PA, USA).

Lipid quality indexes were calculated following Ulbricht and Southgate [45] formulae: atherogenicity index (AI) = [C12:0 + (4 × C14:0) + C16:0]/(Σ *n*-6 PUFA + Σ *n*-3PUFA + Σ MUFA); thrombogenicity index (TI) = (C14:0 + C16:0 + C18:0)/[0.5 × Σ MUFA + 0.5 × Σ *n*-6PUFA + 3× Σ *n*-3PUFA + (Σ *n*-3PUFA/Σ *n*-6PUFA)]

#### 2.5.2. Sensory Analysis

To establish Quantitative Descriptive Analysis (QDA) and evaluate the sensory properties of GSB fillets, six evaluation sessions were established by an internal panel from the research institute, composed of ten evaluators, previously selected and trained according to ISO guidelines [46]. The panelists, together with the panel leader, using the check-all-that-apply (CATA) method [47], established and defined a total of fourteen descriptions of the main sensory attributes of GSB related to odor, appearance, texture, flavor, and residual taste (Table 2) using samples from the fish fed AS and 65SA. The panelists were trained using an unstructured continuous line scale to score the intensity of the sensory attributes.

After filleting, two square pieces (3 cm × 3 cm) were processed from each fish fillet (n = 15). Pieces with skin were cooked in lidded aluminum boxes in an air-heated oven (Compact; Eurofred, Barcelona, Spain) at 105 °C for 10 min. Immediately after cooking, the boxes were covered and served to each panelist in a temperate maintainer (Clatronic International GmbH, Kempen, Germany) to avoid cooling.

The sensory evaluation took place in the test room of SABE (Service of Aquaculture and Biotechnology of High Specialization), which was designed according to ISO guidelines [48]. Each panellist assessed the sensory attribute sets of four blinded samples in random order at six separate sessions during three consecutive days. The assessments were recorded on a continuous scale with anchors presented on a computer screen and ranked from low (value 0) to high intensity (value 100) for each attribute.

#### 2.5.3. Texture Profile Analysis (TPA)

Texture profile analyses were carried out on raw GSB unskinned fillets (n = 15) on days 1 and 4 dph using a TA.XT2 Texture Analyzer (Stable Micro Systems Ltd., Surrey, UK) calibrated with a 5 kg mass. Two square pieces from each fillet were collected (2 cm × 2 cm) above the lateral line. The force–deformation curve was analyzed to determine the hardness, springiness, cohesiveness, gumminess, chewiness, adhesiveness, and resilience parameters according to Ginés et al. [49] using a compression plate with a diameter of 100 mm.

### 2.6. Muscle Histological Studies

#### 2.6.1. Immunohistochemical Analysis

Muscle samples (n = 15) were fixed in 4% buffered paraformaldehyde at 4 °C, dehydrated in an ethanol series, and embedded in paraffin wax. Sections of 3 µm were cut using a semi-automated microtome (Leica Autocut 2055, Leica, Nussloch, Germany) and attached to SuperFrost-Plus slides. After antigen retrieval (high pH, Dako, Glostrup, Denmark), immunohistochemical staining was performed using the EnVision + System (Dako, Denmark). The primary (monoclonal) antibodies used were calpastatin (2G11D6, ThermoScientific-Pierce, Waltham, MA, USA; diluted 1:1000), m-calpain (107-82, ThermoScientific-Invitrogen, Carlsbad, CA, USA; diluted 1:100), µ-calpain (9A4H8D3, ThermoScientific-Invitrogen, Carlsbad, CA, USA; diluted 1:100), dystrophin (Madra 1, Sigma, St. Louis, MO, USA; diluted 1:350), and actin (ThermoScientific-Invitrogen, HHF35; diluted 1:40). Diaminobenzidine (DAB) (Dako, Glostrup, Denmark) was used as a chromogen, and the slides were contrasted with Harris hematoxylin. Negative controls were processed simultaneously and obtained by replacing primary antibodies with a primary antibody diluent. The stained sections were scanned with a MoticEasyScan Pro digital scanner (Motic, Xiamen, China) run using the Motic DS Assistant software (Motic VM V1 Viewer 2.0). Forty sections were analyzed, comprising five antibodies per genotype, diet, and dph. Different pictures were made for muscle types I and II, and the general immunopositivity distribution was described by three trained independent blind observers following semi-quantitative scoring. A representative section was selected from each treatment group and is included in the figure panels.

#### 2.6.2. Transmission Electron Microscopy (TEM)

Muscle samples (n = 15) for the ultrastructural studies of type II muscle were fixed at 4 °C in 2.5% glutaraldehyde, post-fixed in 2% osmium tetroxide and 2% uranyl acetate, dehydrated, and embedded individually in a resin block. Ultrathin (50 nm) sections were photographed and qualitatively evaluated with a Field Emission Scanning Electron Microscope (FESEM; Carl Zeiss Microscopy GmbH, Sigma 300 VP, Jena, Germany) using the transmission mode with STEM in BF detector at the Advanced Confocal and Electron Microscopy Research Service (SIMACE) of the ULPGC. The samples were evaluated for mitochondrial, sarcomere, and connective tissue integrity. Additionally, mitochondrial features such as integrity, location, size, shape, and the presence of intra-mitochondrial granules were revised.

### 2.7. Statistical Analysis

The data obtained are presented as mean ± SD and were statistically analyzed using IBM SPSS Statistics (version 27.0, Armonk, NY, USA, IBM Corp.), testing for normality and homogeneity of variances using Shapiro–Wilk and Levene’s tests, respectively. A General Linear Model (GLM), with diet, genotype, and feed strategy as fixed factors, was used to determine their effects and potential interactions. Differences were considered statistically significant at *p* < 0.05. When significant interactions were detected (*p* < 0.05), a one-way ANOVA was applied to the data to check the differences between treatments using Tukey’s post hoc test [50] (Tukey, 1949). Outliers were identified using boxplot and normality diagnostics in SPSS, and extreme values outside the acceptable range were excluded. The experimental unit for the productive growth parameters (SGR, FCR, and FI) was the tank (n = 3).

For biochemical, textural, and histological post-sacrifice data, a GLM was performed, including post-harvest time as an additional fixed factor. For the sensory analysis data, a GLM was performed using the evaluator as a random factor. Finally, to show the multivariate structure of the sensory evaluation, a principal component analysis (PCA) was conducted in the program Unscrambler X version 10.4 (AspenTech Inc., Bedford, MA, USA).

## 3. Results

### 3.1. Fish Performance

At the end of the experiment, the survival rate of fish was not affected by any of the experimental factors. Table 3 shows the key performance indicators (KPIs) during the experimental period. Significant (*p* < 0.05) differences in growth parameters were found among the dietary treatments, feeding strategies, and genotypes (Table S1 shows effect sizes and confidence intervals). The fish fed the ALT diet showed higher K, SGR, and FI, indicating enhanced growth and intake, whereas those fed the CTRL diet achieved better feed efficiency, as reflected by a lower FCR (Table 3). While the HG fish showed significative better growth parameters compared to the REF genotype, reaching a percentage improvement of approximately 9%, FCR was not significantly affected. Furthermore, the HG fish fed the ALT diet exhibited significantly higher growth compared to the REF fish fed the CTRL diet. The feeding strategy significantly (*p* < 0.05) influenced all the growth parameters except for the FCR. Specifically, increasing the feeding level led to a notable improvement in growth performance and K value, indicating enhanced somatic development with higher FI.

Two significant (*p* < 0.05) interactions between the different experimental factors were also found (Table 3): interaction between diet and genotype for FI, with the REF genotype fed the CTRL diet showing the lowest intake, and interaction between genotype and feeding strategy for final weight, with the highest values being reached in the HG genotype fed AS, while the lowest were shown by the REF genotype fish fed 65AS.

### 3.2. Biochemical Analysis

**Figure 1:**
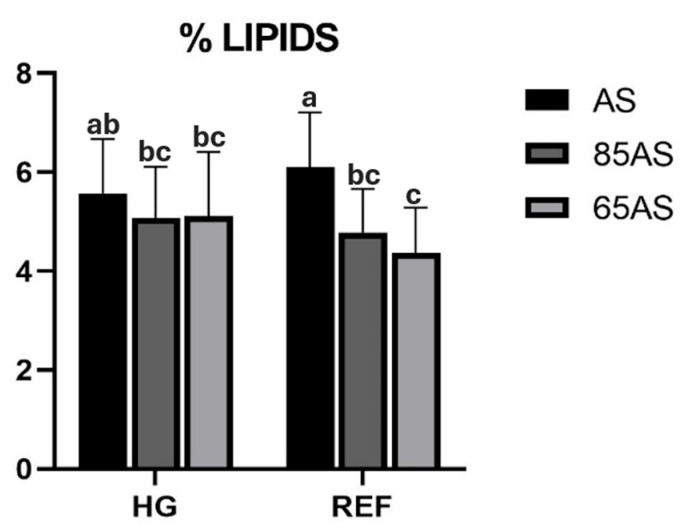
Lipid content (%) of gilthead seabream fillets depending on genotype and feeding strategy. AS: apparent satiation; 85AS: 85% of apparent satiation; 65AS: 65% of apparent satiation; HG: high-growth genotype; REF: reference genotype. n = 15. Values are the mean ± SD. Different letters denote significant differences among the treatments (*p* < 0.05).

Diets and genotypes did not significantly (*p* > 0.05) alter fillet proximate composition (Table 4 and Table S2 show effect sizes and confidence intervals), but feeding strategies significantly influenced the protein, lipid, and moisture content. The fish fed AS increased the relative amount of lipids in the fillet by 18.75% compared to the fish fed 65AS. An interaction between genotype and feeding strategies was also detected for fillet lipid content. While there were no differences in the HG genotype, within the REF genotype, the fish fed the AS strategy achieved a higher percentage of lipids in the fillet (Figure 1). After 4dph, the lipid content of the fillet increased, while the protein content decreased, and was better preserved in the GSBs that had received the ALT diet (Table S3).

Regarding the effect of ice storage days, the amount of protein in muscle decreased significantly after 4 dph, being better preserved in seabream that had received the ALT diet (Table S1).

**Figure 2:**
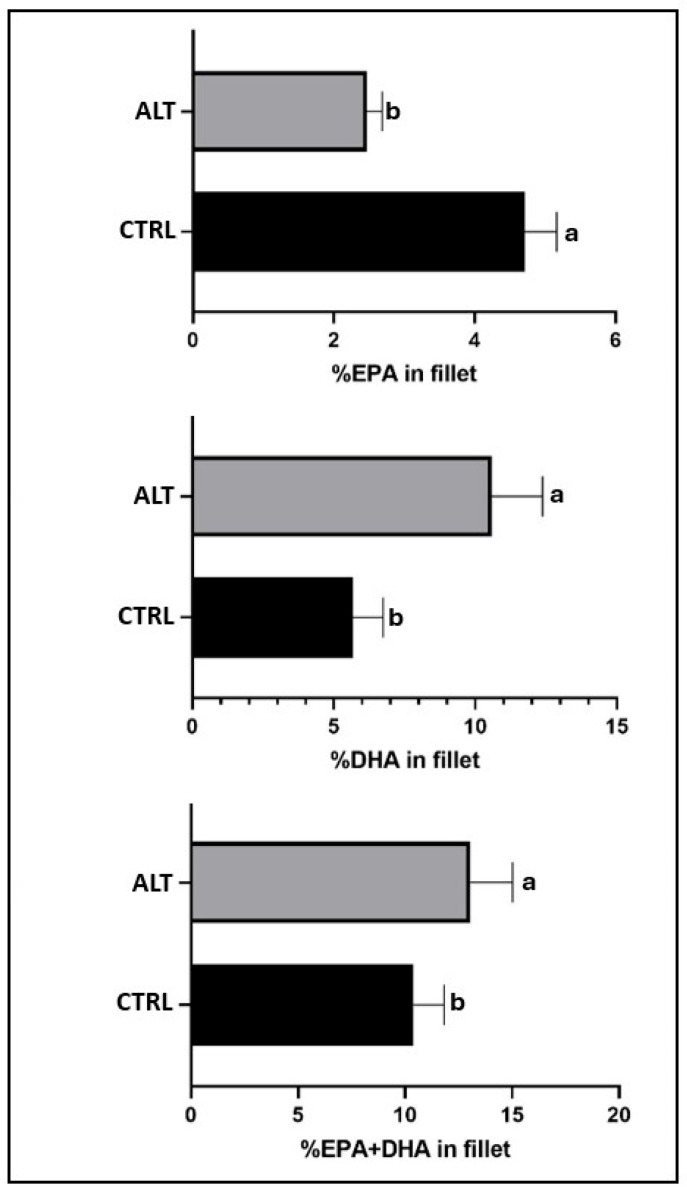
EPA and DHA content (%) of gilthead seabream fillets fed the experimental diets. ALT: alternative diet; CTRL: control diet; DHA: docosahexaenoic acid, 22:6*n*-3; EPA: Eicosapentaenoic acid, 20:5*n*-3. n = 15. Values are the mean ± SD. Different letters denote significant differences among the treatments (*p* < 0.05).

The fatty acid profile of the fillets was significantly (*p* < 0.05) influenced by all the experimental treatments (Table 5 and Table S4 show effect sizes and confidence intervals). The ALT diet elevated the fillet total SAT and total *n*-3LC-PUFA levels, but reduced EPA and MUFA proportions relative to the CTRL diet (Table 5). The fish fed the ALT diet exhibited twice as much docosahexaenoic acid (DHA) and half as much EPA in the fillet compared to those fed the CTRL diet, and vice versa (Figure 2). The REF group had a higher concentration of *n-3*LC-PUFA, while the HG group exhibited more MUFA in the fillet.

**Figure 3:**
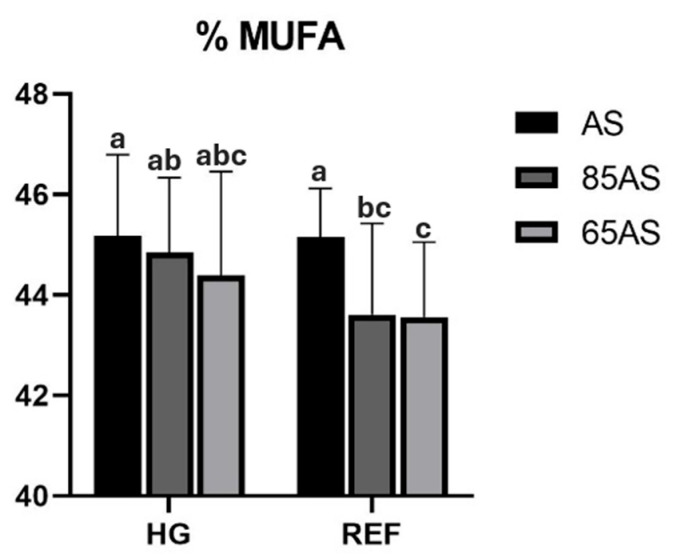
MUFA content (%) of gilthead seabream fillets depending on genotype and feeding strategy. HG: high-growth genotype; REF: reference genotype; AS: apparent satiety; 85AS: 85% of apparent satiety; 65AS: 65% of apparent satiety; MUFA: Monounsaturated fatty acids. n = 15. Values are the mean ± SD. Different letters denote significant differences among the treatments (*p* < 0.05).

Regarding the effect of feeding strategy, GSB fed with dietary restrictions (85AS and 65AS) had a significantly higher percentage of *n-3*LC-PUFA in the fillet than those fed with AS. Conversely, those fed AS had more oleic acid (C18:1*n*-9) than those fed with dietary restrictions. Significant interactions were detected between the different experimental factors (Table 5). An interaction between genotype and feeding strategy was observed for MUFA and oleic acid. Thus, in the HG genotype, the content was similar regardless of the feeding strategy, whereas in the REF genotype, the MUFA content of the AS strategy showed comparable content to that of HG, but significantly higher than those of the 85AS and 65AS strategies (Figure 3).

Concerning the lipid quality indexes, no significant differences were found in the thrombogenicity index (TI) between the experimental factors, whilst the atherogenicity index (AI) was significantly higher (*p* = 0.000) in the fillets from GSBs fed the CTRL diet (0.26) when compared to the ALT diet (0.24).

The ice storage period significantly reduced the levels of *n-3*LC-PUFA after 4 dph, while those of saturated increased (Table S5).

### 3.3. Sensory Analysis

**Figure 4:**
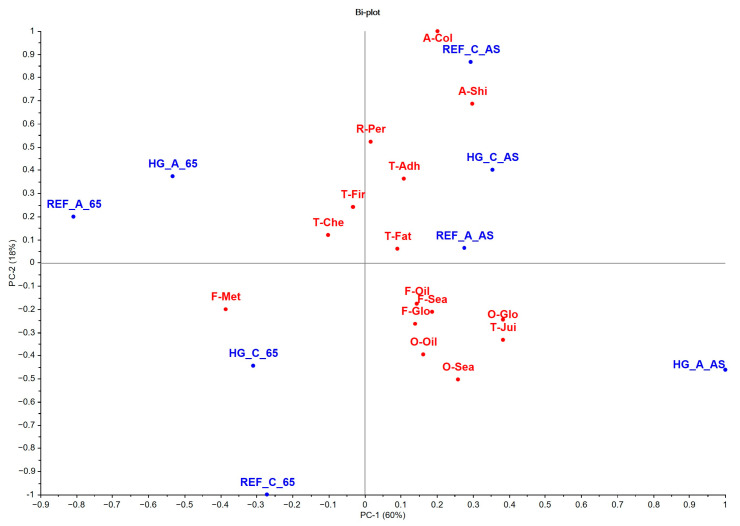
Simultaneous projection of treatments and sensory descriptors of gilthead seabream cooked fillet (n = 30). Sensory descriptors (red color): odor (O)—global intensity (O-Glo), seafood (O-Sea), and oily (O-Oil); flavor (F)—global intensity (F-Glo), seafood (F-Sea), oily (F-Oil), and metallic (F-Met); appearance (A)—white color (A-Col) and shininess (A-Shi); texture (T)—firmness (T-Fir), fatness (T-Fat), adhesiveness (T-Adh), and chewiness (T-Che). Treatments (blue color): high-growth genotype–alternative diet–apparent satiation (HG_A_AS); high-growth genotype–alternative diet–65% of apparent satiation (HG_A_65); high-growth genotype–control diet–apparent satiation (HG_C_AS); high-growth genotype–control diet–65% of apparent satiation (HG_C_65); reference genotype–alternative diet–apparent satiation (REF_A_AS); reference genotype–alternative diet–65% of apparent satiation (REF_A_65); reference genotype–control diet–apparent satiation (REF_C_AS); reference genotype–control diet–65% of apparent satiation (REF_C_65).

**Figure 5:**
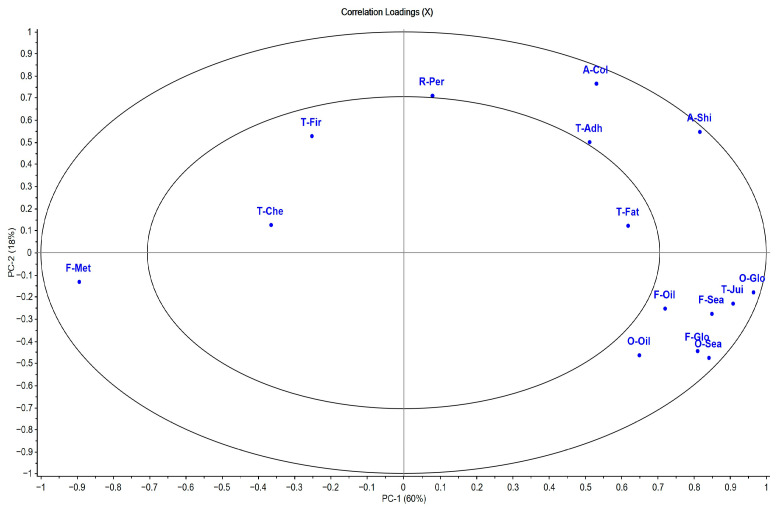
Correlation loadings plot of sensory descriptors of gilthead seabream cooked fillet (n = 30). Sensory descriptors: odor (O)—global intensity (O-Glo), seafood (O-Sea), and oily (O-Oil); flavor (F)—global intensity (F-Glo), seafood (F-Sea), oily (F-Oil), and metallic (F-Met); appearance (A)—white color (A-Col) and shininess (A-Shi); texture (T)—firmness (T-Fir), fatness (T-Fat), adhesiveness (T-Adh), and chewiness (T-Che). The outer ellipse indicates 100% of the explained variance. The inner ellipse indicates 50% of the explained variance.

The principal component analysis (PCA) explained 78% of the total variance. The results of the multivariate analysis were consistent with those obtained by univariate analysis (Figure 4; Table S6). PC1, with 60% of the variance explained, was determined by the global intensity of odor and flavor, oily odor and flavor, fatness and juiciness textures, and fillet shine intensity, associated with the fish that were fed AS. On the contrary, the metallic flavor was located on the left of the graph, associated with the fish fed with the highest dietary restriction of 65AS, with a significant effect (*p* < 0.05) between feeding strategies (Figure 4 and Figure 5; Table S6). PC2 explained 18% of the total variance and was more associated with appearance attributes, such as brightness intensity and whitish color of the fillet (Figure 4 and Figure 5; Table S6).

The univariate analysis revealed that the different diets and genotypes did not significantly (*p* > 0.05) affect the organoleptic properties of the GSB fillet, except for juiciness, which was observed to be higher in HG compared to REF (Table S3). About spoilage, moisture and ash were not affected, while lipid content increased after 4 dph. The protein content decreased significantly after 4 dph, although it was better preserved in fish fed the ALT diet.

### 3.4. Texture Profile Analysis (TPA)

In terms of textural parameters, the fillets from fish fed the ALT diet had significantly (*p* < 0.005) higher hardness and adhesiveness than those from fish fed the CTRL diet. As for the effect of genotype, the REF fish showed higher cohesiveness, gumminess, and resilience than those of the HG genotype. In addition, the 85AS feeding restriction strategy achieved the highest values for almost all the parameters (Table 6). An interaction between diet and feeding strategy was identified for chewiness (Table 6), as GSB fillets fed the ALT diet had significantly higher values than those fed the CTRL diet when the AS strategy was employed. Feeding strategy was the most influential factor in the differences in sensory profile (Table S7 shows effect sizes and confidence intervals).

The ice storage period significantly reduced all the fillet texture parameters after 4 dph (Table S8).

### 3.5. Histological Studies

#### 3.5.1. Immunohistochemistry

Table 7 summarizes the results of the muscle sections labelled using different primary antibodies. At 1 dph, immunohistochemistry of the GSB muscle did not show apparent differences when comparing diets, genotypes, or feeding strategies. In general, type I muscle immunostaining was more pronounced than that of type II muscle, especially at the sarcolemma membrane (Figures S1 and S2). During ice storage, immune reactivity for actin and m-calpain increased, particularly in the REF group (Figure S2). Also at 4 dph, a decrease in fiber-to-fiber adhesion and detachment between the myocommata and myofibers was noted, mainly observed in type I muscle fibers (Figures S1 and S2). Most immunostaining and tissue architecture were preserved after 4 dph, without areas of muscle disintegration.

#### 3.5.2. Transmission Electron Microscopy

**Figure 6:**
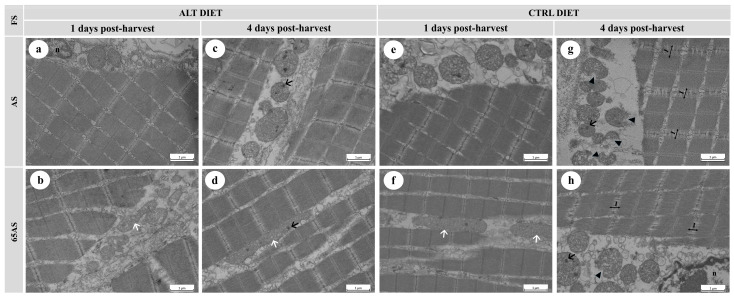
Electron micrographs of longitudinal sections of skeletal muscle from a high-growth genotype (HG) gilthead seabream, showing ultrastructural features stored at 4 °C for 1 and 4 days post-harvest. n = 15. ALT: alternative diet; CTRL: control diet; FS: feeding strategy; AS: apparent satiety; 65AS: 65% of apparent satiety. Scale bar: 1 μm. (**a**) Muscle from AS-fed fish with the ALT diet after 1 day of ice storage, showing well-preserved muscle fibers, mitochondria, and nuclei (n); (**b**) Muscle from 65AS-fed fish with the ALT diet after 1 day of ice storage, exhibiting elongated mitochondria (white arrow); (**c**) Muscle from AS-fed fish with the ALT diet after 4 days of ice storage, showing mitochondria with dense granules (black arrow); (**d**) Muscle from 65AS-fed fish with the ALT diet after 4 days of ice storage, showing elongated mitochondria (white arrow) and mitochondrial dense granules (black arrow); (**e**) Muscle from AS-fed fish with the CTRL diet after 1 day of ice storage, showing preserved ultrastructure; (**f**) Muscle from 65AS-fed fish with the CTRL diet after 1 day of ice storage, exhibiting elongated mitochondria (white arrow); (**g**) Muscle from AS-fed fish with the CTRL diet after 4 days of ice storage, showing widened I-bands (I) and disrupted Z-discs, mitochondria with dense granules (black arrow), and mitochondrial membrane detachment (black arrowhead); (**h**) Muscle from 65AS-fed fish with the CTRL diet after 4 days of ice storage, displaying widened I-bands (I) and disrupted Z-discs, mitochondria with dense granules (black arrow), and membrane detachment (black arrowhead).

**Figure 7:**
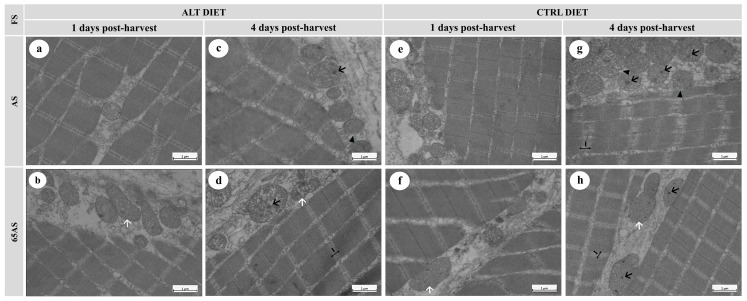
Electron micrographs of longitudinal sections of skeletal muscle from a high-growth genotype (HG) gilthead seabream, showing ultrastructural features stored at 4 °C for 1 and 4 days post-harvest. n = 15. ALT: alternative diet; CTRL: control diet; FS: feeding strategy; AS: apparent satiety; 65AS: 65% of apparent satiety. Scale bar: 1 μm. (**a**) Muscle from AS-fed fish with the ALT diet after 1 day of ice storage, showing well-preserved muscle fibers and mitochondria; (**b**) Muscle from 65AS-fed fish with the ALT diet after 1 day of ice storage, exhibiting elongated mitochondria (white arrow); (**c**) Muscle from AS-fed fish with the ALT diet after 4 days of ice storage, showing mitochondria with dense granules (black arrow), and slight mitochondrial membrane detachment (black arrowhead); (**d**) Muscle from 65AS-fed fish with the ALT diet after 4 days of ice storage, showing elongated mitochondria (white arrow) and mitochondrial dense granules (black arrow); (**e**) Muscle from AS-fed fish with the CTRL diet after 1 day of ice storage, showing preserved ultrastructure; (**f**) Muscle from 65AS-fed fish with the CTRL diet after 1 day of ice storage, exhibiting; (**g**) Muscle from AS-fed fish with the CTRL diet after 4 days of ice storage, showing widened I-bands (I) and disrupted Z-discs, mitochondria with dense granules (black arrow), and mitochondrial membrane detachment (black arrowhead); (**h**) Muscle from 65AS-fed fish with the CTRL diet after 4 days of ice storage, displaying widened I-bands (I) and disrupted Z-discs, elongated mitochondria (white arrow), and mitochondria with dense granules (black arrow).

The principal outcomes observed using TEM are shown in Figure 6 and Figure 7. While diet and genotype had no ultrastructural effects, feeding restrictions (65AS) consistently induced mitochondrial elongation (Figure 6b,d,f and Figure 7b,d,f) and fusion (Figure 7h), likely reflecting metabolic stress.

Ice storage modified both mitochondrial and muscle fiber integrity. At 1 dph, scarce intra-mitochondrial dense granules, slight interfibrillar separation, and detachment of the connective tissue sheathing the muscle fibers were observed (Figure 6b,e,f and Figure 7b,f). After 4 dph, the presence of intra-mitochondrial granules increased (Figure 6c,d and Figure 7c,g,h), often accompanied by mitochondrial membrane disruption (Figure 6g,h) with a slight loss of cristae definition. Ice storage reduced sarcomere integrity, decreased the integrity of the Z-disc, and increased the width of the I-band (Figure 6g,h and Figure 7d,g,h). Ice storage was associated with an increase in interfibrillar separation, including loss of cohesion of the endo- and perimysium with adjacent fibers (Figure 6g,h).

## 4. Discussion

The challenge of gradually replacing FM and FO in aquaculture feeds must be addressed using a holistic research strategy tailored to industry needs [51]. This study aimed to improve knowledge on feed formulations that can be managed and well accepted by genetically selected GSB, and to assess the impact of different feeding strategies in long-term feeding up to commercial size.

The fish performance of GSB was not uniform, as FCR and growth were better with feeding CTRL diet, but SGR, FI, and K with the ALT diet. Different studies have shown that the full or partial replacement of FM or FO independently with alternative ingredients is feasible without negatively affecting fish growth performance, with better results observed when combining multiple alternative ingredients [11,17,18,52,53,54]. Carvalho et al. [8] highlighted, in GSB diets, that replacing FO with a combination of microalgae oil (AO), poultry oil (PO), and vegetable oil (VO) with 15% FM resulted in growth and SGR efficiency similar to the control diet with FO but growth performance decline accompanying the reduced FM percentage.

Research on replacing FM and FO with new and innovative ingredients is still in its early stages. Although several meta-analyses have investigated the replacement of FM and FO with novel ingredients in aquafeeds, none have assessed the combined use of multiple alternative ingredients within a single dietary strategy [12,15,55,56]. The substitution of FM and FO in commercial diets with alternative ingredients such as IM or single-cell protein and a mixture of PO and AO significantly reduced growth and productive parameters like SGR in short-term GSB studies [25,28]. This reduction is presumably due to alternative diets containing less than 15% FM, substituted by a single protein source, different from a vegetable meal (VM). Meagre (*Argyrosomus regius*) fed an alternative diet of insect meal (IM), microalgae biomass, and oil from tuna canning water showed lower FCR but similar growth performance to that of the control diet, although the FM replacement rate was low [57]. It must be considered that using new protein sources to replace FM may worsen zootechnical performance due to suboptimal amino acid profiles; diets with ingredients like poultry meal have lower apparent digestibility for some amino acids compared to diets with less FM substitution, especially in long-term feeding studies when fish reach commercial size [58,59]. The better growth indicators outcomes for ALT diet could involve enhanced amino acid availability, improved FI, and increased feed palatability [11,58].

Based on the genotype results, although there were no differences in FCR, the HG group achieved its intended improvement objective, showing significantly better growth parameters than the REF genotype (approximately 9%). Previous studies with genetically selected GSB [25,28] or in other species testing selected strains such as European seabass (*Dicentrarchus labrax*) [58,60,61] and rainbow trout (*Oncorhynchus mykiss*) [62] have also demonstrated better growth and productive parameters in the selected genotypes, regardless of dietary treatment. The ALT diet results in the HG group suggest that genetic selection can take advantage of novel dietary formulations using emerging ingredients. Previous short-term feeding trials have demonstrated that growth-based genetic selection, when combined with diets based on plant ingredients [24] or novel ingredients, such as poultry and microalgae oils [25], can enhance growth performance [58,63]. This suggests that genetically selected fish are more capable of adapting their metabolism, which is characterized by increased intestinal plasticity [24,28,58,64]. Specifically, GSB selected for high growth and fed alternative diets have demonstrated increased intestinal length to enhance nutrient absorption [24], a more resilient gut microbiota [64], improved digestive enzyme activity, and higher apparent digestibility coefficients (ADC) [28] in comparison with non-selected fish.

In commercial farms, feed intake at AS is not considered an effective practice [29]. Working in tanks with few fish, even at high densities, the AS hand-feeding protocol contributed to the lack of significant differences in FCR. In accordance with the results of this study, feed restriction has been shown to reduce growth parameters [31,65] even when applied cyclically or based on body weight [30,66]. However, when assessing the involvement of genetic selection, the HG genotype makes the best use of feeding at apparent satiety, while the REF genotype is most penalized with the 65AS strategy.

The fish flesh composition appears to be strongly influenced by dietary ingredients, but other parameters such as feed ration and fish size, as in the present study, affect the proximate composition of the fillet [67]. This feed ration effect has also been described in short-term experiences with GSB feed on novel raw materials and involves genetic selection, where fillet proximal composition was not modified [25]. Feeding strategies affect the body composition of fish, especially lipids stored in the liver, viscera, and muscles, which are the main source of energy for maintenance during periods of starvation or feed restriction [65]. The fish fed AS showed a mean increase of 18.75% in lipids and a concomitant 1.63% reduction in fillet moisture content compared to those fed 65AS, an inverse relationship previously reported [23,67]. In fact, the application of feed restriction strategies decreases the percentage of lipids present in fillets, while the moisture content decreases when ration supply is high [66,68]. Additionally, genotype may modulate these effects, suggesting that HG GSB have a greater ability to conserve lipids in fillets under dietary restriction. In European seabass, differing results have been found regarding lipid content and genetic selection [57,59].

Regarding the decline processes during ice storage, it was not affected by treatments, and the reduction in protein percentage detected at 4 dph was due to the concomitant effect of proteolytic enzyme activity [37,69]. A decrease in sarcoplasmic protein content during the dripping process after protein denaturation [23,70] and variations in the proportion of protein content lead to a higher percentage of lipids.

Diet composition often significantly influences the cellular conformation of the fish, particularly the fatty acid composition [71], as in the present study for all the experimental factors studied. Thus, the higher MUFA content in the fish fed the CTRL diet was related to the palmitoleic acid (C16:1*n*-7) present in vegetable and fish oils [72]. The *n*-3LC-PUFA in the fillets from fish fed the ALT diet showed the connection with the high percentage of DHA provided by AO [73], while the higher EPA content present in FO was accumulated to a greater extent in fish fed the CTRL diet. Despite the higher EPA content in the fillet from the CTRL diet, the overall EPA + DHA content in the fillet remained significantly higher in the fillets of GSB fed with the ALT diet. This is particularly relevant considering the importance from a human nutrition perspective in enhancing the reduction in cardiovascular risk, inflammation, and neurodegenerative diseases [74,75,76]. Thus, per 100 g of fillet, the total mg of EPA + DHA provided by the fish fed the ALT diet was 477 mg, almost 25% more than the 397 mg of the fillets of the fish fed the CTRL diet, although in both cases above 250 mg/day recommended by the European Food Safety Authority [77]. Although cooking can lead to a decline of the *n*-3/*n*-6 ratio, since SAT are relatively heat-stable under typical cooking temperatures and LC-PUFA are more susceptible to degradation due to their higher degree of unsaturation, the use of mild cooking methods, such as steam oven processing, can help preserve the lipid quality of the fillet [35].

Not only does the EPA + DHA content in fish have beneficial health effects, but studying the complete fatty acid profile will provide information on the potential cardiovascular risks derived from the atherogenic and thrombogenic effects of SAT and the protective role of PUFA and MUFA [45]. Thus, the lower atherogenicity index (AI) value detected in the fish fed the ALT diet was due to its higher level of PUFAs, especially *n*-3PUFA, along with a lower content of myristic acid (C14:0). This reflects diet composition, with a higher contribution of myristic acid from fish oil in the CTRL diet. For *n*-3PUFA, although the content in both diets was similar, the higher presence of DHA in the ALT diet due to the inclusion of microalgae oil, along with its selective accumulation [78], causes the fish fed this diet to achieve higher proportions of *n*-3PUFA in the fillet. In the case of thrombogenicity index (TI), no differences have been detected between diets, as the higher PUFA values in fish fed the ALT diet have been counterbalanced by a higher content of stearic acid (C18:0), largely contributed to the diet by PO [79]. The low values obtained for both AI and TI, comparable to those already described for farmed gilthead seabream fillets [80,81], support the reduction in cardiovascular disease risk [82], even though no recommended value has been established by public health organizations [83]. This is likely because the platelet aggregation properties of polar and neutral lipid fractions depend on the fish species and their origin [84].

Fish fed the ALT diet showed improved *n*-3 fatty acid levels without increasing *n*-6 fatty acids, resulting in a significantly higher *n*-3/*n*-6 ratio, addressing a key challenge of replacing marine ingredients like FO with *n*-6-rich alternatives such as PO. The novel ingredients in the ALT diet not only avoid negative impacts on zootechnical performance but also enhance the nutritional value of the fillet for consumers.

The fact that the REF genotype had a higher concentration of *n*-3LC-PUFA, while the HG genotype displayed a greater proportion of MUFA, was also noted in a long-term study in European seabass. In this fish species, the selected genotype exhibited lower levels of *n*-3LC-PUFA [60] or exhibited higher levels of DHA in the fillet compared to a commercial genotype [58], showing that the selective breeding for high growth has both positive and negative impacts on fish fillet quality [25]. In rainbow trout, selective breeding for improved growth has led to changes in the fatty acid composition of the fillet, including a reduction in beneficial unsaturated fatty acids [85]. Moreover, genetic selection can alter the expression of crucial genes involved in PUFA biosynthesis, thereby decreasing their proportion in the fillet [86].

Regarding the feeding strategy effect, the significantly higher percentage of *n*-3LC-PUFA in the fillet at 85AS and 65AS compared to AS has also been described in other species, such as lean strains of Atlantic salmon that accumulate more *n*-3LC-PUFA than fat strains [87]. *n*-3LC-PUFA can be selectively retained using different feeding levels to meet the FA requirements and tissue membrane function [68,88,89]. The C18:1*n*-9 proportion found in the AS group was also described in rainbow trout, establishing that starvation or a reduction in feed ration has been related to a decrease in the MUFA ratio due to the association with the decrease in lipid percentage in the fillet [90]. The interaction between genotype and feeding strategy revealed that the MUFA content, including C18:1*n*-9, in the HG fillets remained consistent regardless of the feeding strategy. In contrast, the GSB REF group fed the 85AS and 65AS strategies showed lower MUFA content than the other groups. This connects with the selected phenotypes’ improved endurance to food restriction, which allows them to retain dietary nutrients more efficiently. Currently, dietary management and genetic selection are the primary tools used to control the muscle fat content in farmed fish [68].

The reduction in the percentage of *n*-3LC-PUFA fatty acids, accompanied by an increase in the percentage of SAT throughout the ice storage, indicates that unsaturated fatty acids are more prone to oxidation due to the greater number of double bonds, a critical issue regarding the fatty acid composition of fish fillet [67]. This oxidative process leads to the formation of compounds such as hydroperoxides and aldehydes, which can compromise the skeletal muscle structure and contribute to changes in fillet texture and overall quality, as reflected in our results [91]. In line with this, Alexi et al. [92] reported similar alterations in GSB, linking them to enzymatic degradation and fatty acid breakdown [23]. Previous studies have shown that unlike wild GSB, the fillet of farmed GSB exhibits minimal variation in fatty acid composition during the ice storage period, regardless of the diet provided. Notably, the *n*-3/*n*-6 ratio remains favorable throughout the shelf life, thereby preserving the cardiovascular health benefits associated with fish consumption [23].

In addition to zootechnical and nutritional aspects, sensory characteristics, including texture as perceived by consumers, play a crucial role in the acceptance and market value of the product. Notably, the sensory analysis results indicated that the experimental diets did not significantly affect the long-term organoleptic properties. However, the diets led to variations in the fillet fatty acid profiles, altering the concentration of volatile aldehydes derived from *n*-3 or *n*-6PUFA [34], and even within the *n*-3PUFA category, between EPA and DHA [93]. However, replacing FO with AO and increasing DHA content have not shown a clear influence on sensory attributes, as previously confirmed in both Atlantic salmon [94] and GSB [95]. Only in diets where the inclusion of AO promotes large differences between EPA and DHA accumulations can sensory differences be detected [96]. About the effect of genotype, the higher perceived juiciness intensity in HG fish is likely related to a higher moisture content in the fillet [97], which is associated with the different growth rates.

Interestingly, the feeding strategy was identified as the factor with the greatest impact on the sensory profile. Differences detected in global odor and flavor intensity, the shiny appearance of the fillet, fatness and juiciness textures, and oily odor and flavor describe variations in fillet fat content [98] of more than 20% in the fish fed AS versus 65SA. While for the degree of fat perception in the mouth during chewing, the relationship with fillet fat seems clear, juiciness is also positively correlated and depends on muscle fat content [80,99] because fish fat gives a soft and succulent, i.e., juicy, mouthfeel. Odor and flavor, assessed in both overall intensity and oiliness, are determined by the volatile products of lipid oxidation [97], with fattier fish more prone to develop these characteristics compared to leaner fish. The other effect of the feeding strategy on sensory perception was a metallic flavor associated with increased dietary restriction (65AS). Considering that lipid oxidation is essential to produce a metallic flavor [100], the higher *n*-3LC-PUFA content in the fillet of fish fed a restricted diet conditioned its higher susceptibility to oxidative rancidity, a phenomenon that may be attributable to the presence of metal ions, which catalyze the oxidation of these fatty acids [100].

Regarding fillet texture properties, flesh hardness can be influenced by reducing the force required for compression when fish meal (FM) is decreased in the diet [101], particularly when the final FM content is below 10% and replaced with vegetable meal (VM) [35]. However, substituting both FM and VM with other animal protein sources, as proposed in the ALT diet, retains and even enhances fillet firmness compared to fillets from the fish fed diets containing only 15% FM. The texture of the fish fillet is dependent on the amino acid ratio of the diet [102], necessitating the supply of amino acids such as hydroxyproline, which is considered conditionally essential in fish [103]. This ensures the correct structure and strength of connective tissue [104], which cannot be guaranteed by a vegetable meal-based diet with low or no hydroxyproline contribution [105]. Replacing soy protein concentrate in the control (CTRL) diet with poultry by-product meal has been effective in improving fillet firmness because the hydroxyproline content provided by poultry by-products is 35–45 times higher than that provided by soy [106].

Growth in fish involves recruitment and hypertrophy of muscle fibers and modifications in muscle cellularity that promote variations in meat texture [107] because connective tissue is relatively more abundant in a muscle with high fiber density, and higher values of texture parameters will be achieved [108]. The HG group, which exhibited significantly greater growth than REF over the same period and at the same age, would have experienced hypertrophy to a greater extent. Because of the lower fiber density, the fillet is less cohesive and requires less force to break it up. A moderate feed ration restriction (85AS) has increased the fillet resistance to deformation compared to AS, supported by a low-fat muscle content. In fact, a significant loss of hardness and thus softening of the flesh has been associated with an increase in muscle fat [109,110], which is negatively correlated with the maximum force to compression [111,112].

Proteolytic activity during ice storage induces structural changes in fish fillets, leading to softening and the loss of freshness and quality [113]. The narrow window studied in the present research, up to 4 dph, did not detect significant differences in filamentous proteins during ice storage, indicating resistance to proteolysis. The increased immunoreactivity of actin and m-calpain observed at 4 dph, particularly pronounced in the REF genotype, may be attributed to the activation of calpain proteases during the early post-mortem storage period, which coincides with the initial stages of muscle degradation in fish [114,115]. Furthermore, calpain activity degrades actin filaments, releasing α-actinin from the myofibrils into the cytoplasm, resulting in the loss of Z-disc integrity [38] and increasing actin detection by specific antibodies. The loss of Z-disc integrity contributes to muscle degradation and results in a reduction in fillet firmness, which may negatively influence consumer acceptance [67]. The differential response of calpastatin to m-calpain and μ-calpain in the REF genotype could indicate the role of calpastatin as an endogenous calpain inhibitor [37] and the same differential effect of the HG group on growth and resilience to feed restriction. In Nile tilapia (*Oreochromis niloticus*), calpains and calpastatin exhibited inversely coordinated expressions in response to starvation [115]. In contrast to our findings on GSB, in which no dietary or gene group differences were observed, the endoproteases µ-calpain, m-calpain, and calpastatin remained unaltered throughout ice storage for up to 10 days [37]. Only desmin showed degradation, but this occurred after more than 4 days of ice storage. Similar results were observed in seabass, with immunopositivity loss only after 8 days of ice storage [35]. In this study, anti-calpastatin immunolabeling was maintained, while anti-dystrophin immunoreactivity disappeared [35]. As in this European seabass study, dietary composition had a marginal impact on myofibrillar or endoprotease antibodies during the storage period. The different reactions of m-calpain and µ-calpain are related to their complementary functions and varying calcium requirements, which differ between fish species [37,114].

Under stress, such as starvation, cells can induce various strategies, including mitochondrial fusion or mitochondrial autophagy (mitophagy) [116,117]. This is compatible with the observations in the 65AS group under feeding restriction, where the ultrastructure study revealed elongated mitochondria involved in plastic processes. Mitochondrial fusion may increase under medium stress levels, providing a protective effect on autophagic turnover under nutrient deprivation conditions, inhibiting the onset of apoptosis [118], and determining cell differentiation [119]. Mitophagy combats stress by directly eliminating mitochondria, allowing the replacement of vital macromolecular precursors, including amino acids, sugars, and fatty acids [116]. This result, first time described in fish, agrees with several studies in other species as mouse fibers, where mitochondria are significantly elongated and fused shortly after food restriction or starvation [117,118,120].

Ice storage influences both the mitochondrial and fibrillar integrity. Mitochondrial dense granules, amplified at 4 dph, are intracellular accumulations of calcium deposits, generally in the form of calcium phosphate, within the mitochondrial matrix [121]. The process of mitochondrial granule formation and mitochondrial membrane rupture may be interconnected phenomena. Under conditions of cellular stress, mitochondria accumulate excess calcium by forming dense granules; however, a calcium overload can induce mitochondrial permeability, resulting in the loss of mitochondrial membrane potential and the release of mitochondrial contents into the cytosol [122]. This process activates proteases, such as calpains, which degrade structural proteins and contribute to the softening of fish flesh during the postmortem period [114,123]. These results are consistent with previous studies in GSB and European seabass, where dense granules and a decrease in mitochondrial membrane density were observed after approximately 4 days of ice storage [33,124,125] increased after 6–7 days of ice storage.

Concerning the structural degradation of the fibers, at 1 dph, there was a slight detachment of the connective tissue from the fibers and an increase in interfibrillar separation. Rigor mortis is known to end after 12–36 h post-mortem due to the proteolytic activity of both calpains and lysosomal cathepsins, involved in the early degradation of muscle fibers observed in GSB [33]. Previous findings on GSB and seabass reported that while no changes were observed in the myofibrils, part of the sarcolemma began to detach, and the interfibrillar spaces increased after 1 day of ice storage [33,126]. By 4 dph, these changes were found more frequently along the tissue. The alteration of the sarcomeres evidenced a loss of the integrity of the Z discs along with an increase in the amplitude of the I band. Some of these alterations could already be observed from day 3 in ice in the GSB muscle [124], with significant structural damage identified in the fibers and myofilaments on days 5–6 of ice storage. These changes included fiber and endomysium detachment, as well as a decrease in the density of the Z discs and a barely identifiable I band [33,69,124]. In European seabass muscle [126], similar outcomes were described, whereas in rainbow trout, alterations were recorded at the fibrillar level after 7 days of ice storage, concomitant with the loss of fillet texture [127]. Although proteolytic events appear to be common across different fish species, there are intra- and interspecific variations in the level of muscle degradation [33,126]. In addition, differences due to both intrinsic and extrinsic factors affecting enzymes and proteins [38] are present.

## 5. Conclusions

This study demonstrates that replacing half of the fishmeal in diets with a mix of insect, poultry, feather, and porcine blood meals, along with substituting fish oil entirely with microalgae, poultry, and salmon by-product oils, is feasible for long-term gilthead seabream farming. This alternative diet improves nutritional quality for consumers by increasing EPA + DHA levels, the *n*-3/*n*-6 ratio, and fillet hardness. HG gilthead seabreams showed better growth performance and benefited more from the ALT diet than the REF fish group. The fiber morphological studies were only affected by feeding strategies at the ultrastructural level. The 85AS feeding strategy optimized fillet quality by preventing excess lipid accumulation that impaired texture, seen in the AS feeding strategy, and avoiding stress-induced fibrillar changes, like mitochondrial elongation observed in the 65AS feeding strategy. The HG fish were more resilient to dietary restrictions, conserving lipids and MUFA better than REF fish. Combining genetic selection, moderate dietary restriction, and innovative diets can boost aquaculture sustainability and product quality during long-term feeding.

## Figures and Tables

**Table 1:** Ingredients, proximate and fatty acid composition of the experimental diets (ALT: alternative diet; CTRL: control diet).

	Diets	
Ingredients (%)	CTRL	ALT
Fishmeal Super Prime	15.00	7.50
Feathermeal hydrolysate		7.50
Porcine blood meal		5.00
Poultry meal		10.00
Worm meal (*Tenebrio molitor*)		7.50
Aminopro NT70—*C. glutamicum*		7.50
Soy protein concentrate	16.00	
Wheat gluten	13.60	3.00
Corn gluten meal	7.00	3.00
Soybean meal 48	6.00	5.80
Wheat meal	14.23	15.63
Faba beans (low tannins)	8.00	8.00
Fish oil	7.00	
Salmon oil		4.00
Algae oil (Veramaris^®^) ^1^		3.30
Rapeseed oil	7.70	3.90
Poultry oil		2.85
Vitamin and mineral premix ^2^	1.00	1.00
Proximate composition (% feed)	CTRL	ALT
Crude protein	43.0	46.0
Crude fat	18.0	18.0
Fiber	1.8	1.2
Starch	15.6	14.4
Ash	5.7	6.0
Fatty acids (% feed)	CTRL	ALT
Myristic acid (C14:0)	0.5	0.3
Palmitic acid (C16:0)	1.9	2.4
Stearic acid (C18:0)	0.4	0.5
Oleic acid (C18:1*n*-9)	6.5	6.3
Linoleic acid (LNA, C18:2*n*-6)	2.1	2.6
α-Linolenic acid (ALA, C18:3*n*-3)	0.8	0.8
Arachidonic acid (ARA, C20:4*n*-6)	0.1	0.1
Eicosapentaenoic acid (EPA, C20:5*n*-3)	1.4	0.7
Docosahexaenoic acid (DHA, 22:6*n*-3)	0.8	1.5

^1^ Veramaris algal oil (Veramaris, Delft, The Netherlands). ^2^ Mineral and vitamin premix (Trouw Nutrition, Boxmeer, The Netherlands).

**Table 2:** Sensory attributes for cooked gilthead seabream fillet and attributes definition.

Descriptors	Attributes	Definition
Odor	Global intensity	Global odor intensity
	Seafood	Odor associated with seafood
	Oily	Odor associated with fish oil
Appearance	Color	White color intensity
	Shininess	Intensity of light in the uncut steak
Texture	Firmness	The force required to deform the fillet between the tongue and palate
	Juiciness	Amount of liquid released when the sample is chewed
	Chewiness	Amount of chewing required before swallowing
	Adhesiveness	The degree to which the fillet clings to the teeth during chewing
	Fatness	Degree of perception in the mouth during chewing
Flavor	Global intensity	Global flavor intensity
	Seafood	Flavor associated with seafood
	Oily	Flavor associated with fish oil
	Metalic	Flavor associated with metals
Residual taste	Persistence	Time the mouthfeel lasts

**Table 3:** Growth performance and feed utilization from the high-growth and reference gilthead seabream fed the experimental diets and different feeding strategies.

Diet	Genotype	Feed Strategy	Weight (g)	K	SGR (% Day^−1^)	FCR	FI (g Fish^−1^ Day^−1^)
ALT	HG	AS	285.41 ± 27.85 ^a^	1.72 ± 0.10 ^a^	0.98 ± 0.01 ^ab^	1.24 ± 0.03 ^abc^	1.15 ± 0.03 ^a^
		85AS	235.58 ± 25.30 ^cd^	1.64 ± 0.08 ^b^	0.90 ± 0.01 ^e^	1.25 ± 0.01 ^abc^	0.94 ± 0.01 ^c^
		65AS	192.76 ± 24.72 ^e^	1.53 ± 0.08 ^de^	0.84 ± 0.01 ^f^	1.23 ± 0.04 ^abc^	0.75 ± 0.01 ^e^
	REF	AS	262.97 ± 30.90 ^b^	1.63 ± 0.11 ^b^	0.95 ± 0.01 ^c^	1.29 ± 0.08 ^a^	1.10 ± 0.05 ^ab^
		85AS	225.73 ± 24.65 ^d^	1.58 ± 0.10 ^c^	0.90 ± 0.01 ^e^	1.25 ± 0.02 ^abc^	0.91 ± 0.01 ^cd^
		65AS	177.46 ± 2.77 ^f^	1.48 ± 0.08 ^f^	0.81 ± 0.01 ^g^	1.29 ± 0.04 ^ab^	0.72 ± 0.01 ^ef^
CTRL	HG	AS	293.58 ± 29.51 ^a^	1.69 ± 0.09 ^a^	0.99 ± 0.01 ^a^	1.19 ± 0.01 ^bc^	1.14 ± 0.03 ^a^
		85AS	245.12 ± 20.73 ^c^	1.58 ± 0.10 ^c^	0.93 ± 0.01 ^d^	1.18 ± 0.03 ^c^	0.93 ± 0.02 ^cd^
		65AS	193.62 ± 17.45 ^e^	1.54 ± 0.08 ^cd^	0.84 ± 0.00 ^f^	1.18 ± 0.02 ^c^	0.72 ± 0.1 ^ef^
	REF	AS	271.08 ± 29.82 ^b^	1.64 ± 0.12 ^b^	0.96 ± 0.01 ^bc^	1.19 ± 0.01 ^abc^	1.05 ± 0.01 ^b^
		85AS	228.34 ± 22.23 ^d^	1.53 ± 0.09 ^de^	0.90 ± 0.01 ^e^	1.18 ± 0.00 ^c^	0.87 ± 0.02 ^d^
		65AS	179.51 ± 15.29 ^f^	1.49 ± 0.09 ^ef^	0.81 ± 0.01 ^g^	1.18 ± 0.04 ^c^	0.66 ± 0.02 ^f^
*p*-value		Diet	0.001	0.003	0.007	0.000	0.000
		Genotype	0.000	0.000	0.000	n.s	0.000
		Feed strategy	0.000	0.000	0.000	n.s	0.000
		D × G	n.s	n.s	n.s	n.s	0.036
		D × FS	n.s	n.s	n.s	n.s	n.s
		G × FS	0.036	n.s	n.s	n.s	n.s

Values are expressed in mean ± SD. n = 30 for growth parameters (Weight and K) and n = 3 for productive parameters (SGR, FCR, and FI). Different letters denote significant differences among the treatments for a specific interaction (*p* < 0.05). n.s: not significant. ALT: alternative diet; CTRL: control diet; HG: high-growth genotype; REF: reference genotype; AS: apparent satiety; 85AS: 85% of apparent satiety; 65AS: 65% of apparent satiety. D × G: diet–genotype interaction; D × FS: diet–feeding strategy interaction; G × FS: genotype–feeding strategy interaction.

**Table 4:** Biochemical composition of fillets from the high-growth and reference gilthead seabream fed the experimental diets and different feeding strategies at 1 day post-harvest.

Diet	Genotype	Feed Strategy	Protein	Ash	Lipids	Moisture
ALT	HG	AS	20.58 ± 0.39 ^ab^	1.52 ± 0.09	5.62 ± 1.11 ^abc^	72.64 ± 1.14 ^ab^
		85AS	20.71 ± 0.52 ^ab^	1.58 ± 0.14	5.16 ± 1.11 ^abc^	73.03 ± 1.03 ^ab^
		65AS	20.88 ± 0.39 ^ab^	1.61 ± 0.11	4.87 ± 0.96 ^abc^	73.41 ± 1.12 ^ab^
	REF	AS	20.50 ± 0.39 ^b^	1.51 ± 0.12	6.21 ± 1.08 ^a^	71.96 ± 1.15 ^b^
		85AS	20.89 ± 0.52 ^ab^	1.56 ± 0.11	4.71 ± 1.07 ^bc^	73.29 ± 1.49 ^ab^
		65AS	21.10 ± 0.43 ^a^	1.56 ± 0.12	4.38 ± 1.00 ^c^	73.74 ± 1.48 ^a^
CTRL	HG	AS	20.67 ± 0.36 ^ab^	1.53 ± 0.09	5.52 ± 1.12 ^abc^	72.61 ± 1.36 ^ab^
		85AS	20.92 ± 0.51 ^ab^	1.53 ± 0.21	4.92 ± 0.96 ^abc^	73.13 ± 0.90 ^ab^
		65AS	20.51 ± 0.72 ^b^	1.55 ± 0.10	5.36 ± 1.56 ^abc^	73.11 ± 1.28 ^ab^
	REF	AS	20.74 ± 0.43 ^ab^	1.53 ± 0.10	5.99 ± 1.16 ^ab^	72.05 ± 1.29 ^b^
		85AS	20.96 ± 0.35 ^ab^	1.52 ± 0.13	4.88 ± 0.53 ^abc^	73.25 ± 0.82 ^ab^
		65AS	20.86 ± 0.42 ^ab^	1.59 ± 0.13	4.35 ± 0.85 ^c^	73.71 ± 1.33 ^a^
*p*-value		Diet	n.s	n.s	n.s	n.s
		Genotype	n.s	n.s	n.s	n.s
		Feed strategy	0.009	0.049	0.000	0.000
		D × G	n.s	n.s	n.s	n.s
		D × FS	0.010	n.s	n.s	n.s
		G × FS	n.s	n.s	0.006	n.s

Values are expressed in mean ± SD. n = 15. Different letters denote significant differences among the treatments for a specific interaction (*p* < 0.05). n.s: not significant. ALT: alternative diet; CTRL: control diet; HG: high-growth genotype; REF: reference genotype; AS: apparent satiety; 85AS: 85% of apparent satiety; 65AS: 65% of apparent satiety. D × G: diet–genotype interaction; D × FS: diet–feeding strategy interaction; G × FS: genotype–feeding strategy interaction.

**Table 5:** Fatty acid composition of fillets from the high-growth and reference gilthead seabream fed the experimental diets and different feeding strategies at 1 day post-harvest.

Diet	Genotype	Feed Strategy	Saturated	MUFA	*n* -3	*n* -6	*n* -3LC-PUFA	*n* -3/*n*-6	C18:1*n*-9	EPA	DHA
ALT	HG	AS	19.84 ± 0.76 ^ab^	44.90 ± 1.90 ^ab^	17.27 ± 2.06 ^bc^	16.54 ± 0.39 ^bc^	13.89 ± 2.10 ^bcd^	1.04 ± 0.12 ^b^	37.18 ± 1.67 ^ab^	2.43 ± 0.27 ^c^	9.58 ± 1.74 ^b^
		85AS	20.17 ± 1.03 ^ab^	43.89 ± 2.14 ^abc^	17.81 ± 1.81 ^abc^	16.59 ± 0.62 ^abc^	14.43 ± 2.01 ^abcd^	1.07 ± 0.12 ^ab^	35.99 ± 2.54 ^abc^	2.49 ± 0.20 ^c^	10.17 ± 1.82 ^b^
		65AS	19.80 ± 1.41 ^ab^	43.01 ± 2.09 ^bc^	18.83 ± 1.59 ^ab^	16.84 ± 0.28 ^ab^	15.46 ± 1.69 ^ab^	1.12 ± 0.10 ^ab^	36.11 ± 1.60 a^bc^	2.51 ± 0.21 ^c^	11.13 ± 1.47 ^ab^
	REF	AS	19.81 ± 0.64 ^ab^	44.86 ± 0.71 ^ab^	17.29 ± 1.08 ^bc^	16.57 ± 0.22 ^bc^	13.83 ± 1.16 ^bcd^	1.04 ± 0.07 ^b^	37.60 ± 0.69 ^a^	2.33 ± 0.13 ^c^	9.64 ± 0.99 ^b^
		85AS	20.02 ± 1.56 ^ab^	42.12 ± 2.35 ^c^	19.79 ± 2.25 ^a^	16.61 ± 0.26 ^abc^	16.56 ± 2.33 ^a^	1.19 ± 0.14 ^a^	34.77 ± 2.01 ^c^	2.63 ± 0.19 ^c^	12.03 ± 2.12 ^a^
		65AS	20.99 ± 1.58 ^a^	42.20 ± 2.23 ^c^	18.16 ± 1.43 ^abc^	17.13 ± 0.68 ^a^	15.01 ± 1.48 ^abc^	1.06 ± 0.09 ^ab^	35.38 ± 2.04 ^abc^	2.43 ± 0.17 ^c^	10.89 ± 1.36 ^ab^
CTRL	HG	AS	19.92 ± 1.00 ^ab^	45.64 ± 1.44 ^a^	16.32 ± 1.35 ^c^	15.93 ± 0.59 ^d^	12.43 ± 1.31 ^d^	1.03 ± 0.09 ^b^	35.13 ± 2.00 ^bc^	4.54 ± 0.40 ^b^	5.12 ± 0.73 ^c^
		85AS	18.98 ± 1.12 ^b^	45.49 ± 1.37 ^a^	17.00 ± 1.22 ^bc^	16.34 ± 0.32 ^bcd^	12.95 ± 1.18 ^cd^	1.04 ± 0.09 ^b^	35.68 ± 0.73 ^abc^	4.71 ± 0.43 ^ab^	5.45 ± 0.72 ^c^
		65AS	19.69 ± 1.24 ^ab^	45.38 ± 1.86 ^a^	16.30 ± 1.76 ^c^	16.48 ± 0.36 ^bc^	12.51 ± 1.82 ^d^	0.99 ± 0.12 ^b^	34.35 ± 1.85 ^c^	4.50 ± 0.50 ^b^	5.37 ± 1.22 ^c^
	REF	AS	18.83 ± 0.83 ^b^	45.60 ± 1.17 ^a^	17.21 ± 1.09 ^bc^	16.21 ± 0.36 ^cd^	13.24 ± 1.23 ^cd^	1.06 ± 0.07 ^ab^	35.87 ± 1.00 ^abc^	4.77 ± 0.18 ^ab^	5.45 ± 0.78 ^c^
		85AS	19.52 ± 1.02 ^ab^	44.36 ± 1.52 ^abc^	17.71 ± 1.42 ^abc^	16.16 ± 0.36 ^cd^	13.75 ± 1.47 ^bcd^	1.10 ± 0.09 ^ab^	34.51 ± 1.52 ^c^	5.00 ± 0.40 ^a^	6.01 ± 0.96 ^c^
		65AS	19.35 ± 1.21 ^b^	43.97 ± 1.78 ^abc^	17.95 ± 1.85 ^abc^	16.59 ± 0.40 ^abc^	14.16 ± 1.80 ^bcd^	1.08 ± 0.11 ^ab^	34.50 ± 1.26 ^c^	4.74 ± 0.55 ^ab^	6.51 ± 1.13 ^c^
*p*-value		Diet	0.000	0.000	0.000	0.000	0.000	0.012	0.000	0.000	0.000
		Genotype	n.s	0.004	0.003	n.s	0.003	0.009	n.s	0.038	0.008
		Feed Strategy	n.s	0.000	0.000	0.000	0.002	0.021	0.000	0.014	0.000
		D × G	n.s	n.s	n.s	n.s	n.s	n.s	n.s	0.003	n.s
		D × FS	n.s	n.s	n.s	n.s	n.s	n.s	n.s	n.s	n.s
		G × FS	n.s	0.049	n.s	n.s	n.s	n.s	0.010	n.s	n.s

Values are expressed in mean ± SD. n = 15. Different letters denote significant differences among the treatments for a specific interaction (*p* < 0.05). n.s: not significant. ALT: alternative diet; CTRL: control diet; HG: high-growth genotype; REF: reference genotype; AS: apparent satiety; 85AS: 85% of apparent satiety; 65AS: 65% of apparent satiety. D × G: diet–genotype interaction; D × FS: diet–feeding strategy interaction; G × FS: genotype–feeding strategy interaction.

**Table 6:** Texture properties of fillets from the high-growth and reference gilthead seabream fed the experimental diets and different feeding strategies at 1 day post-harvest.

Diet	Genotype	Feed Strategy	Hardness	Adhesiveness	Springiness	Cohesiveness	Gumminess	Chewiness	Resilience
ALT	HG	AS	69.13 ± 8.64 ^cd^	−0.33 ± 0.10 ^bcde^	0.44 ± 0.08	0.22 ± 0.02	14.98 ± 2.27 ^bcd^	6.64 ± 1.56 ^bcd^	0.09 ± 0.01
		85AS	81.85 ± 13.53 ^ab^	−0.45 ± 0.17 ^a^	0.46 ± 0.06	0.21 ± 0.02	17.47 ± 3.91 ^ab^	8.04 ± 1.88 ^ab^	0.11 ± 0.04
		65AS	71.07 ± 10.72 ^cd^	−0.40 ± 0.17 ^abcd^	0.41 ± 0.08	0.21 ± 0.02	14.90 ± 2.76 ^bcd^	5.95 ± 1.10 ^cd^	0.09 ± 0.01
	REF	AS	72.06 ± 10.90 ^bc^	−0.40 ± 0.17 ^abcd^	0.45 ± 0.07	0.23 ± 0.03	16.31 ± 3.42 ^abc^	7.28 ± 1.90 ^abc^	0.11 ± 0.02
		85AS	85.47 ± 18.13 ^a^	−0.48 ± 0.15 ^a^	0.47 ± 0.08	0.22 ± 0.02	18.90 ± 4.79 ^a^	8.71 ± 2.22 ^a^	0.11 ± 0.04
		65AS	70.44 ± 9.99 ^cd^	−0.31 ± 0.13 ^cde^	0.41 ± 0.09	0.21 ± 0.02	15.07 ± 2.63 ^bcd^	6.37 ± 1.88 ^cd^	0.10 ± 0.01
CTRL	HG	AS	60.83 ± 9.55 ^d^	−0.31 ± 0.11 ^cde^	0.44 ± 0.07	0.21 ± 0.02	12.99 ± 2.40 ^d^	5.75 ± 1.27 ^d^	0.09 ± 0.01
		85AS	83.18 ± 11.64 ^a^	−0.43 ± 0.14 ^ab^	0.46 ± 0.08	0.22 ± 0.02	17.96 ± 3.23 ^a^	8.14 ± 1.69 ^a^	0.09 ± 0.01
		65AS	68.54 ± 8.47 ^cd^	−0.29 ± 0.09 ^e^	0.44 ± 0.08	0.22 ± 0.02	15.07 ± 2.16 ^bcd^	6.59 ± 1.31 ^bcd^	0.10 ± 0.01
	REF	AS	65.68 ± 9.53 ^cd^	−0.41 ± 0.14 a^bc^	0.43 ± 0.07	0.22 ± 0.02	14.25 ± 2.59 ^cd^	6.32 ± 1.65 ^cd^	0.09 ± 0.01
		85AS	82.82 ± 15.23 ^a^	−0.46 ± 0.13 ^a^	0.45 ± 0.06	0.22 ± 0.02	18.20 ± 4.06 ^a^	8.15 ± 2.17 ^a^	0.11 ± 0.03
		65AS	67.89 ± 12.03 ^cd^	−0.28 ± 0.13 ^de^	0.42 ± 0.09	0.22 ± 0.01	15.12 ± 3.05 ^bcd^	6.30 ± 1.50 ^cd^	0.09 ± 0.01
*p*-value		Diet	0.007	0.049	n.s	n.s	n.s	n.s	n.s
		Genotype	n.s	n.s	n.s	0.012	0.031	n.s	0.028
		Feed Strategy	0.000	0.000	0.001	n.s	0.000	0.000	0.008
		D × G	n.s	n.s	n.s	n.s	n.s	n.s	n.s
		D × FS	n.s	n.s	n.s	0.006	n.s	0.032	n.s
		G × FS	n.s	0.001	n.s	n.s	n.s	n.s	n.s

Values are expressed in mean ± SD. n = 30. Different letters denote significant differences among the treatments for a specific interaction (*p* < 0.05). n.s: not significant. ALT: alternative diet; CTRL: control diet; HG: high-growth genotype; REF: reference genotype; AS: apparent satiety; 85AS: 85% of apparent satiety; 65AS: 65% of apparent satiety. D × G: diet–genotype interaction; D × FS: diet–feeding strategy interaction; G × FS: genotype–feeding strategy interaction.

**Table 7:** Semi-quantitative immunostaining scoring of cytoskeletal proteins and endoprotease antibodies in gilthead seabream muscle sections, ice-stored for 1 and 4 days post-harvest.

Tx	AB	Type I		Type II	
		D1	D4	D1	D4
HG-ALT	Anti-actin	++/−	+++/−	++/−	++/−
	Anti-calpastatin	++	+++/−	+	+
	Anti-m-calpain	++	++	+	++/−
	Anti-µ-calpain	++/−	++	+/−	+/−
	Anti-dystrophin	+	++/−	−	+/−
HG-CTRL	Anti-actin	++	++	+	+
	Anti-calpastatin	+++/−	+++/−	++/−	++/−
	Anti-m-calpain	++	++	+	+
	Anti-µ-calpain	++	++	+/−	+/−
	Anti-dystrophin	+/−	+	−	+/−
REF-ALT	Anti-actin	++/−	++	+	++/−
	Anti-calpastatin	−	+/−	+/−	+
	Anti-m-calpain	++	+++/−	+	+
	Anti-µ-calpain	+/−	+/−	−	−
	Anti-dystrophin	++/−	++/−	−	+/−
REF-CTRL	Anti-actin	+	+++/−	+	++/−
	Anti-calpastatin	+/−	+/−	+	+
	Anti-m-calpain	+++/−	+++	+	+
	Anti-µ-calpain	+	+/−	−	−
	Anti-dystrophin	++/−	+	−	+/−

Immunoreactivity scoring positive for the molecule examined: +++, >75%; +++/−, 60–75%; ++, 45–60%; ++/−, 30–45%; +, 15–30%; +/−, <15%; −, negative. n = 15. Tx: treatment; AB: antibody; ALT: alternative diet; CTRL: control diet; HG: high-growth genotype; REF: reference genotype.

## Data Availability

The raw data supporting the conclusions of this article will be made available by the authors upon request.

## Supplementary Materials

The following supporting information can be downloaded at https://www.mdpi.com/article/10.3390/ani15131913/s1, Table S1: Confidence intervals and effect size of growth performance and feed utilization from high-growth and reference gilthead seabream fed the experimental diets and different feeding strategies; Table S2: Confidence intervals and effect size of fillet biochemical composition from high-growth and low-growth gilthead seabream fed the experimental diets and different feeding strategies at 1 days post-harvest; Table S3: Biochemical composition of fillets from high-growth and low-growth gilthead seabream fed the experimental diets and different feeding strategies at 4 days post-harvest; Table S4: Confidence intervals and effect size of fillet fatty acid composition from high-growth and low-growth gilthead seabream fed the experimental diets and different feeding strategies at 1 days post-harvest; Table S5: Fatty acid composition of fillets from high-growth and low-growth gilthead seabream fed the experimental diets and different feeding strategies at 4 days post-harvest; Table S6: Sensory attributes of cooked fillets from high-growth and low-growth gilthead seabream fed the experimental diets and different feeding strategies; Table S7: Confidence intervals and effect of fillets texture properties from high-growth and low-growth gilthead seabream fed the experimental diets and different feeding strategies at 1 days post-harvest; Table S8: Texture properties of fillets from high-growth and low-growth gilthead seabream fed the experimental diets and different feeding strategies at 4 days post-harvest; Figure S1: Changes in immunostaining patterns for actin, calpastatin, m-calpain, µ-calpain, and dystrophin cytoskeletal proteins in cross-sections of high-growth genotype (HG) gilthead seabream muscle stored at 4 °C during 1 and 4 days post-harvest. Tx: treatment; AB: antibody. Scale bar: Type I muscle: 60 µm; Type II muscle: 100 µm; Figure S2: Changes in immunostaining patterns for actin, calpastatin, m-calpain, µ-calpain, and dystrophin cytoskeletal proteins in cross-sections of reference genotype (REF) gilthead seabream muscle stored at 4 °C during 1 and 4 days post-harvest. Tx: treatment; AB: antibody. Scale bar: Type I muscle: 60 µm; Type II muscle: 100 µm.
