# Supplementary material for: Impact of Alternative Feed Ingredients and Feeding Strategies on Growth, Muscle Morphology, and Fillet Quality of Genetically Selected Gilthead Seabream (Sparus aurata) in a Long-Term Feeding Trial
Source: Animals (Basel). 2025 Jun 28;15(13):1913. doi: 10.3390/ani15131913 (PMC12248742; doi:10.3390/ani15131913)
Supplement: Supplementary file 1 [file animals-15-01913-s001.zip › animals-3697637-supplementary.pdf]

SUPPLEMENTARY MATERIAL

Table S1. Confidence intervals and effect size of growth performance and feed utilization from high growth and reference gilthead seabream fed the experimental diets and different feeding strategies.

Diet	Genotype	Feed strategy	Weight (g)	K	SGR (% day-1)	FCR	FI (g fish-1 day-1)
ALT	HG	AS	279.508 / 291.310	1.696 / 1.738	0.962 / 0.991	1.174 / 1.299	1.084 / 1.216
		85AS	230.219 / 240.934	1618 / 1.653	0.889 / 0.918	1.232 / 1.261	0.929 / 0.958
		65AS	187.523 / 198.000	1.509 / 1.545	0.915 / 0.965	1.123 / 1.330	0.732 / 0.761
	REF	AS	256.380 / 269.551	1.610 / 1.655	0.925 / 0.975	1.093 / 1.494	0.971 / 1.229
		85AS	220.570 / 230.897	1.562 / 1.603	0.882 / 0.911	1.209 / 1.285	0.892 / 0.921
		65AS	173.086 / 181.835	1.459 / 1.493	0.785 / 0.835	1.182 / 1.398	0.702 / 0.731
CTRL	HG	AS	287.327 / 299.832	1.674 / 1.711	0.972 / 1.001	1.172 / 1.201	1.061 / 1.213
		85AS	240.671 / 249.562	1.557 / 1.601	0.912 / 0.941	1.105 / 1.248	0.876 / 0.991
		65AS	189.967 / 17.278	1.529 / 1.560	0.840 / 0.840	1.145 / 1.221	0.709 / 0.738
	REF	AS	263.380 / 278.787	1.607 / 1.669	0.892 / 1.019	1.063 / 1.317	0.982 / 1.109
		85AS	223.571 / 233.103	1.507 / 1.545	0.875 / 0.925	1.180 / 1.180	0.829 / 0.905
		65AS	176.285 / 182.727	1.468 / 1.505	0.799 / 0.828	1.071 / 1.288	0.612 / 0.715
Effect size		Diet	0.011	0.009	0.280	0,625	0.444
		Genotype	0.106	0.093	0.809	n.s	0.702
		Feed strategy	0.703	0.335	0.989	n.s	0.987
		D × G	n.s	n.s	n.s	n.s	0.178
		D × FS	n.s	n.s	n.s	n.s	n.s
		G × FS	0.007	n.s	n.s	n.s	n.s

Confidence interval values are expressed as means (Lower limit/Upper limit). n=15. n.s: not significant. ALT: alternative diet; CTRL: control diet; HG: high growth genotype; REF: reference genotype; AS: apparent satiety; 85AS: 85% of apparent satiety; 65AS: 65% of apparent satiety. D × G: diet-genotype interaction; D × FS: diet-feeding strategy interaction; G × FS: genotype-feeding strategy interaction.

Table S2. Confidence intervals and effect size of fillet biochemical composition from high growth and low growth gilthead seabream fed the experimental diets and different feeding strategies at 1 days post-harvest

Diet	Genotype	Feed Strategy	Protein	Ash	Lipids	Moisture
ALT	HG	AS	20.362 / 20.793	1.467 / 1.570	5.001 / 6.231	72.011 / 73.272
		85AS	20.423 / 21.000	1.498 / 1.658	4.548 / 5.780	72.464 / 73.606
		65AS	20.667 / 21.095	1.550 / 1.669	4.336 / 5.397	72.796 / 74.032
	REF	AS	20.281 / 20.716	1.474 / 1.586	5.616 / 6.809	71.321 / 72.599
		85AS	20.601 / 21.177	1.450 / 1.594	4.118 / 5.303	72.468 / 74.113
		65AS	20.861 / 21.333	1.521 / 1.664	3.830 / 4.934	72.922 / 74.560
CTRL	HG	AS	20.468 / 20.863	1.478 / 1.577	4.896 / 6.142	71.861 / 73.368
		85AS	20.558 / 21.290	1.379 / 1.685	4.232 / 5.604	72.488 / 73.773
		65AS	20.107 / 20.910	1.495 / 1.601	4.496 / 6.225	72.405 / 73.822
	REF	AS	20.503 / 20.977	1.449 / 1.578	5.348 / 6.633	71.338 / 72.765
		85AS	20.714 / 21.210	1.183 / 1.645	4.505 / 5.257	72.656 / 73.836
		65AS	20.630 / 21.097	1.495 / 1.626	3.882 / 4.819	72.977 / 74.449
Effect size		Diet	n.s	n.s	n.s	n.s
		Genotype	n.s	n.s	n.s	n.s
		Feed Strategy	0.058	0.037	0.108	0.155
		D × G	n.s	n.s	n.s	n.s
		D × FS	0.056	n.s	n.s	n.s
		G × FS	n.s	n.s	0.063	n.s

Confidence interval values are expressed as means (Lower limit/Upper limit). n=15. n.s: not significant. ALT: alternative diet; CTRL: control diet; HG: high growth genotype; REF: reference genotype; AS: apparent satiety; 85AS: 85% of apparent satiety; 65AS: 65% of apparent satiety. D × G: diet-genotype interaction; D × FS: diet-feeding strategy interaction; G × FS: genotype-feeding strategy interaction.

Table S3. Biochemical composition of fillets from high growth and low growth gilthead seabream fed the experimental diets and different feeding strategies at 4 days post-harvest

Diet	Genotype	Feed Strategy	Protein	Ash	Lipids	Moisture
ALT	HG	AS	20.20±0.31^{abc}	1.55±0.26	5.76±1.16^{bcd}	73.29±1.02^{abc}
		85AS	20.04±0.58^{abcd}	1.49±0.26	5.91±1.75^{bc}	72.44±1.64^{cde}
		65AS	20.33±0.42^{ab}	1.59±0.23	4.45±0.68^{d}	74.28±0.81^{a}
	REF	AS	20.38±0.44^{a}	1.46±0.10	5.92±1.56^{abc}	72.99±1.57^{abcd}
		85AS	19.76±0.49^{bcd}	1.56±0.13	6.03±0.99^{abc}	72.46±1.30^{cde}
		65AS	20.16±0.47^{abc}	1.62±0.61	5.04±0.78^{cd}	74.05±1.19^{abc}
CTRL	HG	AS	19.65±0.58^{cd}	1.63±0.50	5.80±1.24^{bcd}	72.75±1.74^{abcde}
		85AS	20.14±0.56^{abc}	1.47±0.17	6.55±1.22^{ab}	71.53±1.24^{de}
		65AS	19.85±0.30^{abcd}	1.44±0.13	4.99±0.88^{cd}	74.23±1.19^{ab}
	REF	AS	19.54±0.40^{d}	1.45±0.10	7.35±1.04^{a}	71.19±1.27^{e}
		85AS	20.20±0.50^{abc}	1.41±0.20	6.11±0.80^{abc}	72.61±0.92^{bcde}
		65AS	19.88±0.46^{abcd}	1.49±0.12	5.25±1.01^{bcd}	73.82±1.21^{abc}
p-value		Diet	0.000	n.s	0.006	0.005
		Genotype	n.s	n.s	0.037	n.s
		Feed Strategy	n.s	n.s	0.000	0.000
		D × G	n.s	n.s	n.s	n.s
		D × FS	0.000	n.s	n.s	n.s
		G × FS	n.s	n.s	n.s	0.010

Values are expressed in mean ± SD. n= 15. Different letters denote significant differences among the treatments for a specific interaction ($p<0.05$). n.s: not significant. ALT: alternative diet; CTRL: control diet; HG: high growth genotype; REF: reference genotype; AS: apparent satiety; 85AS: 85% of apparent satiety; 65AS: 65% of apparent satiety. D × G: diet-genotype interaction; D × FS: diet-feeding strategy interaction; G × FS: genotype-feeding strategy interaction.

Table S4. Confidence intervals and effect size of fillets fatty acid composition from high growth and low growth gilthead seabream fed the experimental diets and different feeding strategies at 1 days post-harvest

Diet	Genotype	Feed Strategy	Saturated	MUFA	n-3	n-6	n-3LC-PUFA	n-3/n-6	C18:1n-9	EPA	DHA
ALT	HG	AS	19.422 / 20.267	43.673 / 45.636	16.127 / 18.412	16.320 / 16.750	12.723 / 15.048	0.976 / 1.113	36.257 / 38.111	2.352 / 2.595	8.617 / 10.541
		85AS	19.5734 / 20.762	43.477 / 45.189	16.760 / 18.852	16.591 / 16.901	13.264 / 15.588	1.007 / 1.142	35.414 / 37.571	2.378 / 2.609	9.122 / 11.219
		65AS	19.020 / 20.585	42.312 / 44.342	17.946 / 19.704	16.688 / 17.000	14.521 / 16.391	1.062 / 1.175	35.229 / 36.990	2.399 / 2.628	10.320 / 11.950
	REF	AS	19.453 / 20.166	44.461 / 45.250	16.691 / 17.889	16.444 / 16.691	13.18714.472	1.004 / 1.0848	37.222 / 37.982	2.256 / 2.401	9.091 / 10.185
		85AS	19.621 / 20.971	41.428 / 43.592	18.541 / 21.037	16.469 / 16.760	15.271 / 17.848	1.116 / 1.266	34.250 / 36.005	2.56 / 2.740	10.855 / 13.205
		65AS	20.229 / 21.012	42.054 / 43.356	17.343 / 19.051	16.806 / 17.124	14.108 / 15.882	1.025 / 1.123	35.211 / 36.457	2.341 / 2.457	10.107 / 11.735
CTRL	HG	AS	19.365 / 20.477	44.845 / 46.444	15.571 / 17.064	15.763 / 16.292	11.699 / 13.151	0.974 / 1.077	34.862 / 36.234	4.315 / 4.758	4.714 / 5.523
		85AS	18.172 / 19.779	44.517 / 46.473	16.124 / 17.873	16.119 / 16.570	12.110 / 13.797	0.987 / 1.094	35.161 / 36.207	4.406 / 5.023	4.936 / 5.964
		65AS	19.006 / 20.380	44.347 / 46.409	15.327 / 17.279	16.278 / 16.681	11.496 / 13.516	0.925 / 1.055	33.325 / 35.379	4.3323 / 4.817	4.818 / 6.171
	REF	AS	18.235 / 19.428	44.766 / 46.439	16.435 / 17.988	15.949 / 16.466	12.365 / 14.119	1.011 / 1.114	335.161 / 16.587	4.641 / 4.894	4.891 / 6.006
		85AS	18.960 / 20.090	44.163 / 45.198	16.929 / 18.498	15.965 / 16.361	12.935 / 14.566	1.045 / 1.149	33.670 / 35.358	4.771 / 5.068	5.492 / 6.103
		65AS	18.680 / 20.024	42.649 / 45.255	16.929 / 18.980	16.370 / 16.812	13.165 / 15.159	1.020 / 1.146	33.803 / 35.198	4.563 / 5.089	5.880 / 7.133
Effect size		Diet	0.108	0.218	0.107	0.208	0.205	0.040	0.189	0.941	0.783
		Genotype	n.s	0.054	0.055	n.s	0.057	0.042	n.s	0.028	0.045
		Feed Strategy	n.s	0.124	0.071	0.199	0.075	0.048	0.157	0.054	0.116
		D × G	n.s	n.s	n.s	n.s	n.s	n.s	n.s	0.003	n.s
		D × FS	n.s	n.s	n.s	n.s	n.s	n.s	n.s	n.s	n.s
		G × FS	n.s	0.040	n.s	n.s	n.s	n.s	0.059	n.s	n.s

Confidence interval values are expressed as means (Lower limit/Upper limit). n=15. n.s: not significant. ALT: alternative diet; CTRL: control diet; HG: high growth genotype; REF: reference genotype; AS: apparent satiety; 85AS: 85% of apparent satiety; 65AS: 65% of apparent satiety. D × G: diet-genotype interaction; D × FS: diet-feeding strategy interaction; G × FS: genotype-feeding strategy interaction.

Table S5. Fatty acid composition of fillets from high growth and low growth gilthead seabream fed the experimental diets and different feeding strategies at 4 days post-harvest

Diet	Genotype	Feed Strategy	Saturated	MUFA	n-3	n-6	n-3LC-PUFA	EPA	DHA	n-3/n-6	C18:1n-9
ALT	HG	AS	22.65±4.78	45.95±2.18^{abc}	14.26±3.12^{bc}	16.20±1.32	11.41±2.65^{abc}	1.93±0.38^{c}	7.91±1.95^{ab}	0.96±0.10	38.29±2.12
		85AS	20.40±3.20	45.08±4.10^{abc}	16.69±3.39^{abc}	16.33±0.89	13.53±3.11^{ab}	2.40±0.30^{c}	9.54±2.42^{a}	1.06±0.14	37.09±3.37
		65AS	23.70±6.20	41.92±5.09^{c}	16.67±3.99^{abc}	14.71±3.15	13.45±3.74^{ab}	2.07±0.50^{c}	9.76±2.83^{a}	1.10±0.08	35.35±2.65
	REF	AS	20.81±2.76	44.41±2.45^{abc}	16.49±1.39^{abc}	16.64±0.55	13.22±1.46^{ab}	2.27±0.23^{c}	9.14±1.13^{a}	1.00±0.08	36.09±2.16
		85AS	18.93±2.92	45.39±1.69^{abc}	17.07±3.21^{ab}	16.45±0.98	13.77±3.10^{ab}	2.50±0.44^{c}	9.51±2.48^{a}	1.04±0.18	35.53±2.83
		65AS	21.42±3.21	45.17±1.92^{abc}	15.70±3.40^{abc}	15.50±2.26	12.76±3.17^{abc}	2.12±0.37^{c}	9.07±2.48^{a}	1.01±0.16	35.47±2.86
CTRL	HG	AS	19.68±1.89	46.74±1.70^{ab}	15.42±1.53^{abc}	15.95±0.49	11.56±1.34^{abc}	4.14±0.43^{a}	4.82±0.74^{c}	0.97±0.10	34.94±1.73
		85AS	19.55±1.30	48.80±2.35^{a}	14.11±2.17^{bc}	15.81±0.81	10.25±1.79^{bc}	4.01±0.50^{ab}	3.99±0.87^{c}	0.90±0.13	33.93±7.29
		65AS	21.80±3.72	45.96±2.52^{abc}	16.46±2.42^{abc}	15.65±1.27	12.49±2.01^{abc}	4.25±1.10^{a}	5.02±1.17^{c}	1.02±0.10	34.47±2.12
	REF	AS	23.84±7.63	46.33±2.02^{abc}	12.68±4.35c	15.73±0.99	9.41±3.40^{c}	3.33±0.27^{b}	3.94±1.32^{c}	0.93±0.10	33.88±4.19
		85AS	20.88±5.03	43.20±5.99^{bc}	16.61±1.74^{abc}	15.54±1.59	12.56±1.47^{abc}	4.60±0.63^{a}	5.23±0.61^{c}	1.07±0.08	34.27±5.51
		65AS	18.17±3.34	45.68±1.12^{abc}	18.39±2.12^{a}	16.48±0.55	14.34±2.06^{a}	4.77±0.49^{a}	6.41±1.24^{bc}	1.12±0.11	35.36±2.12
p-value		Diet	n.s	0.12	n.s	n.s	0.006	0.000	0.000	n.s	0.006
		Genotype	n.s	n.s	n.s	n.s	n.s	n.s	n.s	n.s	n.s
		Feed Strategy	n.s	n.s	0.003	n.s	0.005	0.000	0.019	0.000	n.s
		D × G	n.s	0.017	n.s	n.s	n.s	n.s	n.s	0.014	n.s
		D × FS	n.s	n.s	0.012	0.022	0.048	0.004	n.s	n.s	n.s
		G × FS	n.s	0.013	n.s	n.s	n.s	0.034	n.s	n.s	n.s

Values are expressed in mean ± SD. n= 15. Different letters denote significant differences among the treatments for a specific interaction (p<0.05). n.s: not significant. ALT: alternative diet; CTRL: control diet; HG: high growth genotype; REF: reference genotype; AS: apparent satiety; 85AS: 85% of apparent satiety; 65AS: 65% of apparent satiety. D × G: diet-genotype interaction; D × FS: diet-feeding strategy interaction; G × FS: genotype-feeding strategy interaction.

Table S6. Sensory attributes of cooked fillets from high growth and low growth gilthead seabream fed the experimental diets and different feeding strategies

Diet	Genotype	Feeding Strategy	O-Global	O-Seafood	O-Oily	A-Colour	A-Shininess	T-Firmness	T-Juiciness	T-Chewiness	T-Adhesiveness	T-Fatness	F-Global	F-Seafood	F-Oily	F-Metalic	RT-Persistence
ALT	HG	AS	69.93±16.84	57.67±18.70	34.80±24.28	64.34±20.37	60.80±24.95	55.33±19.08	63.52±17.68	45.47±16.78	45.53±17.39	35.27±19.53	68.87±15.36	54.00±18.73	33.60±23.55^{a}	24.73±23.47^{a}	63.33±17.25^{a}
		65AS	61.53±18.86	50.80±18.05	30.27±22.01	63.33±22.36	57.27±26.13	55.87±20.45	53.73±19.14	48.69±17.36	46.00±17.69	32.13±18.23	65.33±15.13	49.27±16.69	30.60±20.20^{a}	33.93±23.50^{a}	64.90±15.76^{a}
	REF	AS	67.00±18.88	55.27±20.87	31.73±19.35	63.87±19.20	59.67±21.50	55.53±19.12	57.24±16.93	52.20±18.48	45.93±17.80	35.59±18.24	66.13±15.80	51.20±18.76	34.13±19.85^{a}	28.20±20.97^{a}	63.72±15.39^{a}
		65S	59.73±21.26	49.93±19.70	29.17±21.74	61.33±19.24	54.73±25.33	57.40±19.93	53.47±18.62	50.73±18.59	43.40±19.24	34.47±21.46	64.13±17.24	48.07±18.30	30.40±21.20^{a}	33.60±27.32^{a}	64.90±1516^{a}
CTRL	HG	AS	66.20±16.97	53.07±18.83	29.59±21.16	64.60±20.28	60.13±25.11	55.00±21.16	61.00±15.53	52.40±20.39	46.80±18.59	34.73±18.61	67.10±15.20	50.87±15.75	34.27±21.52^{a}	27.72±23.19^{a}	66.87±13.98^{a}
		65AS	62.69±21.93	52.14±24.31	29.47±19.71	61.33±23.04	54.73±28.38	53.60±21.91	55.27±17.42	49.45±19.09	41.60±18.01	33.66±17.56	66.53±15.69	52.07±20.85	30.93±18.59^{a}	28.73±25.39^{a}	60.97±18.60^{a}
	REF	AS	65.80±19.88	52.70±23.37	31.80±20.78	70.00±15.91	60.40±24.08	55.90±18.47	56.70±20.85	47.80±20.21	45.00±17.12	34.00±18.78	65.20±17.72	52.10±18.51	31.20±21.29^{a}	27.60±25.27^{a}	64.70±16.74^{a}
		65S	65.13±17.19	54.73±20.67	33.40±24.62	59.67±20.84	53.40±24.38	54.67±23.70	57.52±14.05	50.00±18.35	43.45±16.66	32.76±19.52	66.20±16.60	50.93±17.55	33.00±22.27^{a}	34.07±28.81^{a}	62.67±19.31^{a}
p-value		Diet	n.s	n.s	n.s	n.s	n.s	n.s	n.s	n.s	n.s	n.s	n.s	n.s	n.s	n.s	n.s
		Genotype	n.s	n.s	n.s	n.s	n.s	n.s	0.043	n.s	n.s	n.s	n.s	n.s	n.s	n.s	n.s
		Feeding Strategy	0.048	n.s	n.s	0.003	0.012	n.s	0.017	n.s	n.s	n.s	0.040	n.s	0.016	0.027	n.s
		D × G	n.s	n.s	0.025	n.s	n.s	n.s	n.s	0.035	n.s	n.s	n.s	n.s	n.s	n.s	n.s
		D × FS	0.013	0.019	n.s	n.s	n.s	n.s	n.s	n.s	n.s	n.s	n.s	n.s	n.s	n.s	n.s
		G × FS	n.s	n.s	n.s	n.s	n.s	n.s	n.s	n.s	n.s	n.s	n.s	n.s	n.s	n.s	n.s

Values are expressed in mean ± SD. n=15. Different letters denote significant differences among the treatments for a specific interaction ($p<0.05$). n.s: not significant. ALT: alternative diet; CTRL: control diet; HG: high growth genotype; REF: reference genotype; AS: apparent satiety; 85AS: 85% of apparent satiety; 65AS: 65% of apparent satiety. D × G: diet-genotype interaction; D × FS: diet-feeding strategy interaction; G × FS: genotype-feeding strategy interaction.

Table S7. Confidence intervals and effect size of fillets texture properties from high growth and low growth gilthead seabream fed the experimental diets and different feeding strategies at 1 days post-harvest

Diet	Genotype	Feed Strategy	Hardness	Adhesiveness	Springiness	Cohesiveness	Gumminess	Chewiness	Resilience
ALT	HG	AS	65.848 / 72.420	-0.3688 / -0.290	0.413 / 0.472	0.211 / 0.224	14.114 / 15.840	6.049 / 7.218	0.088 / 0.097
		85AS	76.797 / 86.898	-0.517 / -0.388	0.441 / 0.488	0.205 / 0.220	16.006 / 18.928	7.344 / 8.746	0.092 / 0.121
		65AS	67.067 / 75.073	-0.46- / -0.338	0.377 / 0.435	0.204 / 0.216	13.865 / 15.929	5.537 / 6.358	0.087 / 0.095
	REF	AS	67.987 / 76.129	-0.463 / -0.338	0.421 / 0.471	0.216 / 0.236	15.035 / 17.586	6.568 / 7.984	0.098 / 0.113
		85AS	78.705 / 92.243	-0.533 / -0.420	0.438 / 0.496	0.213 / 0.227	17.110 / 20.690	7.886 / 9.543	0.090 / 0.121
		65AS	66.570 / 74.315	-0.360 / -0.260	0.377 / 0.448	0.207 / 0.223	14.059 / 16.095	5.656 / 7.085	0.093 / 0.100
CTRL	HG	AS	57.266 / 64.402	-0.350 / -0.268	0.420 / 0.469	0.206 / 0.219	12.090 / 13.883	5.270 / 6.221	0.085-0.093
		85AS	78.831 / 87.520	-0.483 / -0.382	0.426 / 0.488	0.209 / 0.222	16.751 / 19.161	7.506 / 8.771	0.091 / 0.099
		65AS	65.373 / 71.699	-0.327 / -0.261	0.411 / 0.469	0.212 / 0.228	14.260 / 15.876	6.105 / 7.081	0.092 / 0.102
	REF	AS	61.211 / 70.143	-0.477 / -0.352	0.398 / 0.470	0.206 / 0.225	13.043 / 15.464	5.526 / 7.119	0.088 / 0.101
		85AS	77.139 / 88.509	-0.512 / -0.418	0.424 / 0.471	0.211 / 0.228	16.687 / 19.721	7.341 / 8.959	0.093 / 0.118
		65AS	63.397 / 72.383	-0.328 / -0.230	0.388 / 0.455	0.218 / 0.228	13.985 / 16.262	5.742 / 6.863	0.091 / 0.098
Effect size		Diet	0.022	0.011	n.s	n.s	n.s	n.s	n.s
		Genotype	n.s	n.s	n.s	0.019	0.014	n.s	0.014
		Feed Strategy	0.275	0.151	0.044	n.s	0.197	0.219	0.029
		D × G	n.s	n.s	n.s	n.s	n.s	n.s	n.s
		D × FS	n.s	n.s	n.s	0.030	n.s	0.020	n.s
		G × FS	n.s	0.043	n.s	n.s	n.s	n.s	n.s

Confidence interval values are expressed as means (Lower limit/Upper limit). n=15. n.s: not significant. ALT: alternative diet; CTRL: control diet; HG: high growth genotype; REF: reference genotype; AS: apparent satiety; 85AS: 85% of apparent satiety; 65AS: 65% of apparent satiety. D × G: diet-genotype interaction; D × FS: diet-feeding strategy interaction; G × FS: genotype-feeding strategy interaction.

Table S8. Texture properties of fillets from high growth and low growth gilthead seabream fed the experimental diets and different feeding strategies at 4 days post-harvest

Diet	Genotype	Feed Strategy	Hardness	Adhesiveness	Springiness	Cohesiveness	Gumminess	Chewiness	Resilience
ALT	HG	AS	47.75±8.46^{c}	-0.32±0.12^{ab}	0.40±0.07^{bc}	0.19±0.02^{d}	9.11±2.23^{e}	3.67±1.17^{e}	0.08±0.02^{bc}
		85AS	59.68±12.51^{ab}	-0.36±0.16^{ab}	0.48±0.08^{a}	0.23±0.02^{a}	13.46±3.08^{ab}	6.34±1.62^{a}	0.09±0.02^{a}
		65AS	53.37±15.25^{bc}	-0.35±0.10^{ab}	0.37±0.08^{c}	0.20±0.02^{bcd}	10.45±3.65^{cde}	4.03±1.48^{de}	0.08±0.02^{bc}
	REF	AS	48.88±11.56^{c}	-0.29±0.10^{b}	0.41±0.08^{abc}	0.20±0.02^{bcd}	9.91±2.86^{de}	4.09±1.42^{cde}	0.08±0.01^{abc}
		85AS	62.87±10.78^{ab}	-0.35±0.12^{ab}	0.45±0.09^{ab}	0.22±0.02^{a}	14.08±2.36^{a}	6.37±1.70^{a}	0.09±0.02^{a}
		65AS	56.55±11.91^{abc}	-0.35±0.11^{ab}	0.38±0.07^{c}	0.20±0.02^{bcd}	11.38±2.98^{bcde}	4.27±1.26^{cde}	0.08±0.02^{abc}
CTRL	HG	AS	53.69±11.63^{abc}	-0.36±0.13^{ab}	0.46±0.08^{ab}	0.22±0.02^{ab}	11.59±2.87^{bcd}	5.31±1.73^{abc}	0.08±0.01^{abc}
		85AS	56.21±12.86^{abc}	-0.38±0.13^{ab}	0.43±0.08^{abc}	0.20±0.01^{bcd}	11.36±2.75^{bcde}	4.74±1.16^{bcde}	0.08±0.01^{abc}
		65AS	63.70±10.09^{a}	-0.37±0.13^{ab}	0.41±0.08^{abc}	0.21±0.01^{abc}	13.58±2.58^{ab}	5.67±1.86^{ab}	0.09±0.02^{abc}
	REF	AS	54.31±13.44^{abc}	-0.33±0.13^{ab}	0.46±0.06^{ab}	0.22±0.02^{a}	12.19±3.27^{abcd}	5.56±1.38^{ab}	0.09±0.01^{abc}
		85AS	59.79±9.97^{ab}	-0.42±0.14^{a}	0.41±0.06^{abc}	0.21±0.02^{abc}	12.49±2.05^{abc}	5.11±0.88^{abcd}	0.09±0.02^{abc}
		65AS	56.12±9.98^{abc}	-0.32±0.12^{ab}	0.42±0.09^{abc}	0.21±0.02^{ab}	12.14±2.65^{abcd}	5.11±1.41^{abcd}	0.09±0.01^{ab}
p-value		Diet	n.s	n.s	n.s	0.002	0.007	0.004	n.s
		Genotype	n.s	n.s	n.s	0.011	n.s	n.s	n.s
		Feed Strategy	0.000	0.008	0.000	0.001	0.000	0.000	n.s
		D × G	n.s	n.s	n.s	n.s	n.s	n.s	n.s
		D × FS	0.005	n.s	0.000	0.000	0.000	0.000	0.000
		G × FS	n.s	n.s	n.s	n.s	n.s	n.s	n.s

Values are expressed in mean ± SD. n= 15. Different letters denote significant differences among the treatments for a specific interaction ($p<0.05$). n.s: not significant. ALT: alternative diet; CTRL: control diet; HG: high growth genotype; REF: reference genotype; AS: apparent satiety; 85AS: 85% of apparent satiety; 65AS: 65% of apparent satiety. D × G: diet-genotype interaction; D × FS: diet-feeding strategy interaction; G × FS: genotype-feeding strategy interaction.

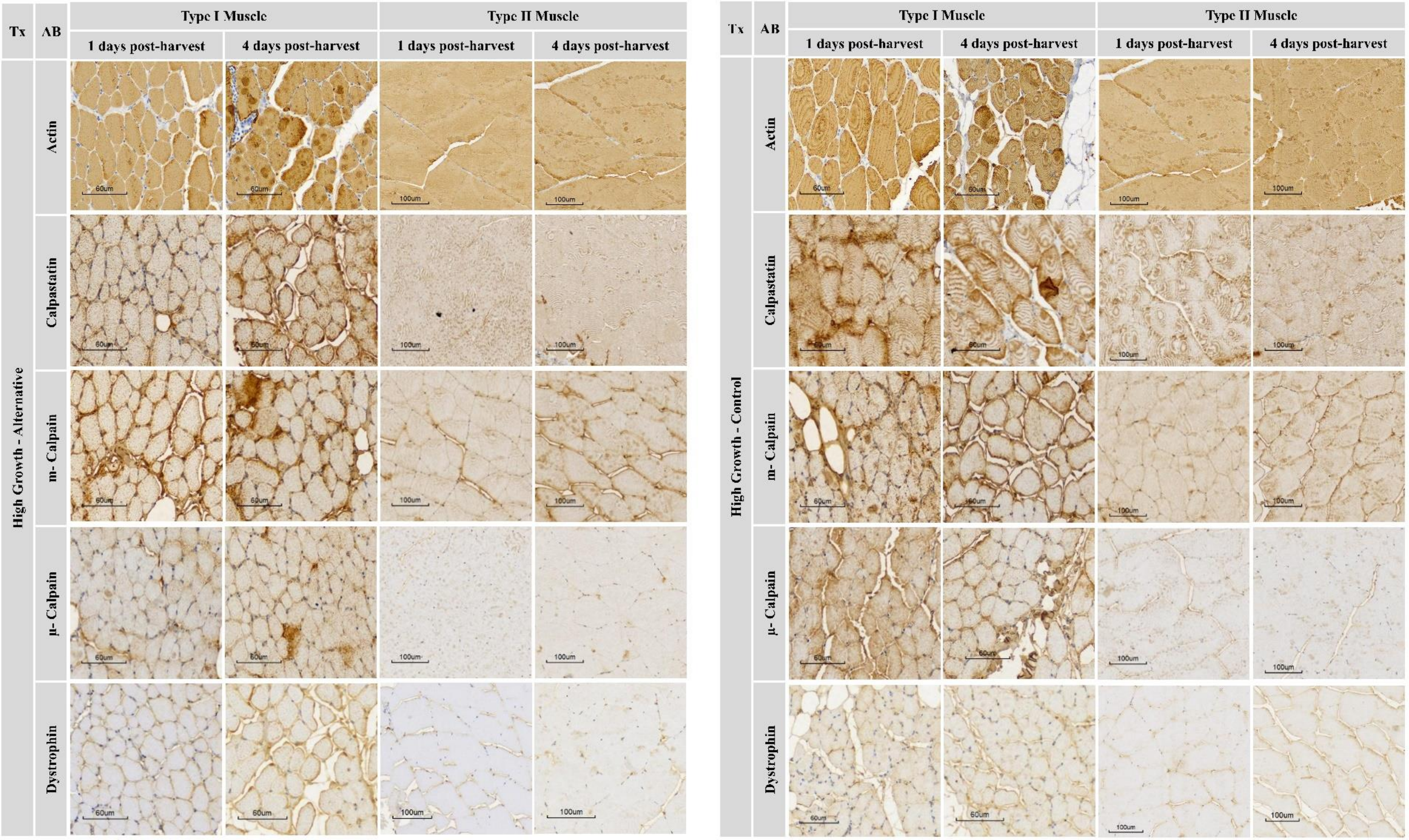

Figure S1. Changes in immunostaining patterns for actin, calpastatin, m-calpain, µ-calpain, and dystrophin cytoskeletal proteins in cross-sections of high growth genotype (HG) gilthead seabream muscle stored at 4ºC during 1 and 4 days post-harvest. n= 15. Tx: treatment; AB: antibody. Scale bar: Type I muscle: 60 µm; Type II muscle: 100 µm.

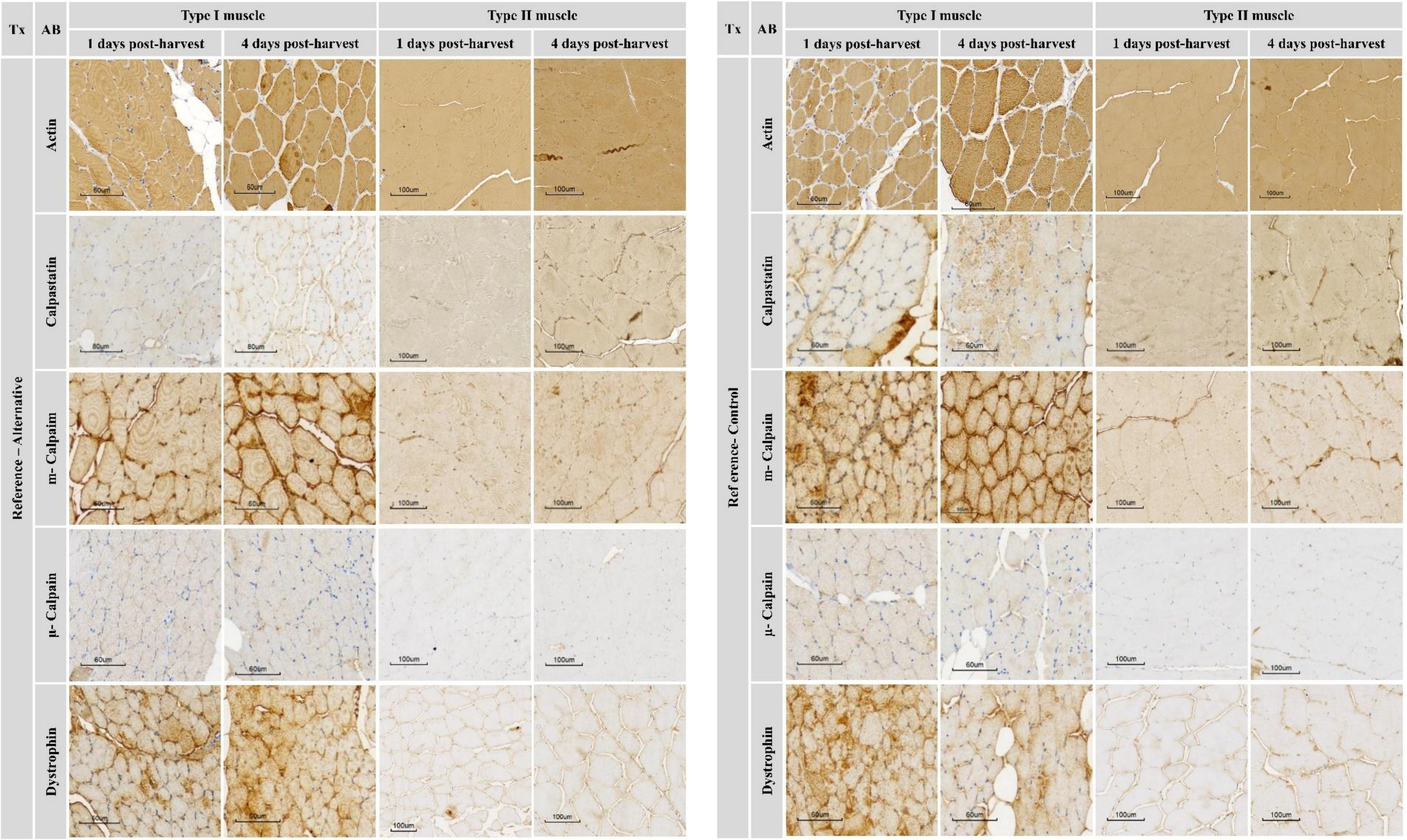

Figure S2. Changes in immunostaining patterns for actin, calpastatin, m-calpain, µ-calpain, and dystrophin cytoskeletal proteins in cross-sections of reference genotype (REF) gilthead seabream muscle stored at 4ºC during 1 and 4 days post-harvest. n= 15. Tx: treatment; AB: antibody. Scale bar: Type I muscle: 60 µm; Type II muscle: 100 µm.
